# Prodrugs Targeting Prostate-Specific Membrane Antigen against Prostate Cancer

**DOI:** 10.1021/acs.jmedchem.4c02626

**Published:** 2025-06-12

**Authors:** Christos Liolios, George Laros, Kyriakos Georgiou, Antonios Kolokouris

**Affiliations:** Laboratory of Medicinal Chemistry, Section of Pharmaceutical Chemistry, Department of Pharmacy, National and Kapodistrian University of Athens (NKUA), Panepistimiopolis−Zografou, 15771 Athens, Greece

## Abstract

Prostate-specific membrane antigen (PSMA) or glutamate carboxypeptidase II (GCPII) is a promising target for metastatic castration-resistant prostate cancer (mCRPC) due to its high expression in prostate cancer cell membranes and low expression in normal tissues. Ligand-targeted drugs (LTDs) targeting PSMA are gaining popularity for their high affinity, specificity, and ease of synthesis. LTDs consist of a small molecule targeting PSMA and carrying the cytotoxic agent to the cancer cells, which is then released through a cleavable linker (enzymatically or chemically cleaved) into the tumor tissue. Several PSMA-targeting LTDs have demonstrated promising results in both *in vitro* and *in vivo* studies, and a few are in clinical trials, showing potential to outperform the unmodified cytotoxic drugs. This article focuses on their structures and preclinical studies while discussing clinical data for a few very promising PSMA-targeting LTDs in detail.

## Graphical abstract

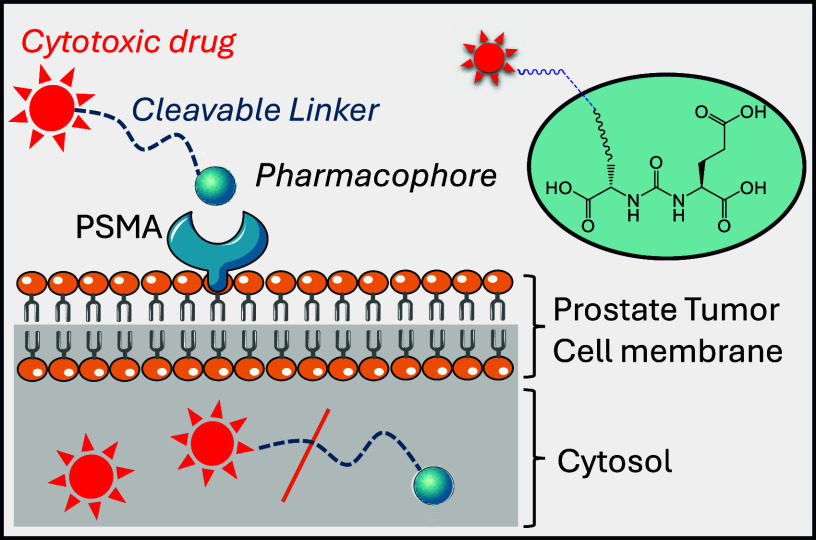

## Brief Statement of the Significance, the Impact, and the Innovation of This Perspective Article

Ligand-targeted prodrugs (LTDs) targeting prostate-specific membrane antigen (PSMA) are a novel groundbreaking drug category, potentially leading to effective treatment strategies and better patient outcomes.

LTDs characteristics:

- High affinity, specificity, and ease of synthesis
- Improved tumor targeting (precision medicine)
- Effective toxophore release (enzymatically or chemically cleaved) inside or in proximity to the tumor (minimization of side effects)

This perspective focuses on their design and structural and biological characteristics, while commenting on the most promising candidates and providing perspectives for their future development.

## Background

Prostate cancer (PCa) has shown the highest incidence rates in cancer diagnosis in males, while being the second most fatal cancer, after lung and bronchus.^1^ Classical chemotherapy against cancer uses potent cytotoxic drugs with a small therapeutic window, which often results in severe side effects.^2^ In addition, tumor cells often develop resistance to these compounds, due to the decreasing drug transport, increasing efflux, and avoiding apoptosis.^3^ Nonspecific treatment such as chemotherapy of PCa (i.e., taxane) or endoradiotherapy (i.e., ^223^RaCl_2_ Xofigo, Bayer) may also result in short remissions; however, at some point resistance of the cancer cells is often observed.^4^ At this stage, durable remissions remain rare, while side effects of the nonspecific treatments due to the killing of normal cells represent a major problem, reflecting the patient’s quality of life. Thus, the need of novel therapeutic approaches is always urgent, for example, new cytotoxic drugs with adequate drug carriers and other selected precision medicine therapies. The high fatality rates of PCa are mainly due to the metastasis of the disease. Metastatic castration-resistant prostate cancer (mCRPC) develops in nearly all PCa patients, who then require additional therapy over time.^4,5^ Over the past decade, the second-generation antiandrogen therapeutics, e.g., apalutamide, darolutamide, and enzalutamide, and chemohormonal therapy have been added in the typical treatment of mCSPC. This addition has significantly improved therapeutic outcomes in men. Nevertheless, approximately 30% of the patients with mCRPC have a 5-year survival rate.^1,4^ The ideal way to maximize safety and efficacy and minimize side effects is to combine the therapeutic toxophore with a targeting moiety, which has a low affinity for healthy cells, but a high affinity for pathological cancer cells.^6^

In *small-molecule drug conjugates (SMDCs)*, a small molecule (pharmacophore) is used for targeting a protein expressed in cancer cells (molecular weight ranging = 1–5 kDa) and delivering the conjugated toxophore in them.^7^ The target protein should be overexpressed in cancer cells compared with the physiological ones, to achieve targeted toxicity. A subcategory of SMDCs is the *ligand-targeted prodrugs (LTDs)*.^6,8,9^ In these bioconjugates, the small molecule pharmacophore is conjugated to a cytotoxic drug through a cleavable linker. At the site of action, the linker is cleaved through a chemical or enzymatic process releasing the cytotoxic drug.^10^

LTDs targeting prostate-specific membrane antigen (PSMA) are a novel groundbreaking drug category potentially leading to effective treatment strategies and better patient outcomes. (Their characteristics have been recently reviewed by Chen et al. 2023.)^11^ However, while PSMA-targeting radiotheranostics, i.e., ^68^Ga/^177^Lu-PSMA-617, have surged ahead, nonradioactive PSMA-targeting drugs (carrying cytotoxic or other payloads, such as G202) are moving more slowly. How do the two categories compare, and what are the hints for the development of PSMA-targeting LTDs from the success story of PSMA radioligands? This article tries to answer these questions while focusing on the PSMA-targeting LTDs. Their design and structural and biological characteristics are analyzed, in addition to the synthetic strategies applied so far and the problems arising during their development. The most promising candidates are distinguished, while some perspectives for LTDs’ future development are being provided.

### Physiology of PSMA/GCPII

Targeting of certain proteins that overexpressed in cancer cells in comparison to normal tissues has been extensively used for receptor-targeted imaging or therapy.^12,13^ The prostate-specific membrane antigen (PSMA)/glutamate carboxypeptidase II (GCPII) is a protein overexpressed in various types of PCa cells, while in healthy humans PSMA/GCPII is localized in prostate (secretory-acinar epithelium) (PSMA expression is increased by 7x times in PCa),^14,15^ nervous system (astrocytes and Schwann cells), kidney (proximal tubules), and small intestine (jejunal brush border membranes) tissues.^16–18^ PSMA/GCPII is a binuclear zinc metallopeptidase (a 2-fold symmetric homodimer) located at the cell surface, which acts as a glutamate carboxypeptidase catalyzing the hydrolytic cleavage of α- or γ-linked glutamates from peptides or small molecules and thus participating in signal transmission via neural pathways and intestinal folate absorption.^16–20^

The natural substrates of PSMA are linked with its two unique enzymatic functions: (i) the *folate hydrolase activity* (PSMA on the surface of the small intestine), which cleaves the terminal glutamates from γ-linked poly glutamates resulting in folic acid and γ-glutamates (folyl-poly-γ-glutamates are essential nutrients), (ii) the *NAALADase activity* (PSMA located in neuronal synapses), which cleaves the terminal glutamate from the neuro-dipeptide, *N*-acetyl-aspartyl-glutamate, (NAAG) resulting to *N*-acetyl aspartate (NAA) and l-glutamate (Figure 1).

**Figure 1:**
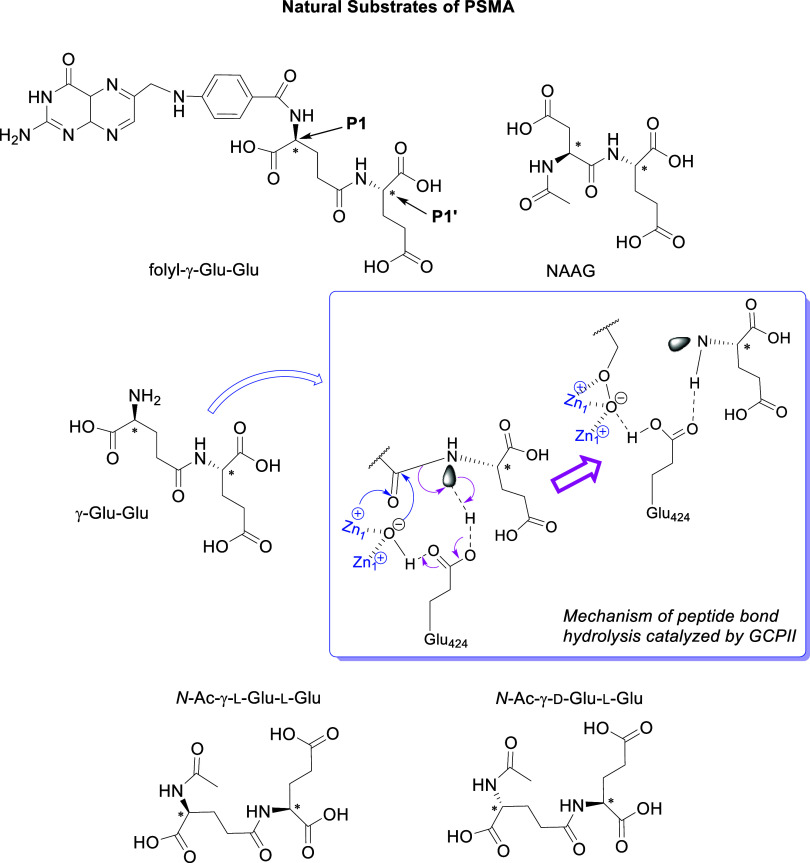
Natural Substrates of PSMA: folyl-γ-Glu (small intestine), NAAG (nervous system), and γ-Glu-Glu and mechanism of peptide bond hydrolysis by GCPII. Position 1 (P1) of the substrate interacts with three (+) charged Arg (Arg534, Arg536, and Arg463), which form the wall of the S1 binding site (arginine patch with affinity for acidic groups placed in P1). The S1′ (pharmacophore) pocket is also positively charged and very sensitive to glutamate residues. Blue square: the mechanism of peptide bond catalysis for P1′ by GCPII.^18^

Crystal structures of PSMA/GCPII with various ligands have provided insight into the essential structural characteristics for their binding.^21^ Interestingly, Position 1 (P1) modifications on the substrate γ-Glu-Glu showed that the addition of d-amino acids, (Ac)-γ-d-Glu-l-Glu, slightly improved the binding affinity to GCPII compared with that of (Ac)-γ-l-Glu-l-Glu. Conversely, the 1-d enantiomers of other natural substrates (NAAG, β-NAAG, and Ac-d-Glu-l-Glu) were hydrolyzed by rhGCPII less efficiently than their 1-l stereochemistry counterparts.^22^

### PSMA/GCPII Binding Site

During the progression of the malignancy, cancer cells begin to express PSMA/GCPII as an insoluble protein formed in the membrane surface and finally overexpress PSMA/GCPII in high-grade and metastatic PCa.^17,23,24^ This form is composed of 750 amino acids, featuring a very short intracellular domain (1–18 amino acids), a transmembrane domain (19–43), and a large extracellular domain (44–750).^16,19^ The extracellular part is responsible for enzymatic protease action. Normal prostate cells express a truncated form of PSMA, PSM’, which lacks the intracellular and transmembrane domains.^12,25^ The X-ray structure of the PSMA/GCPII ectodomain (residues 44–750) was resolved at pH 7 with resolution 3.5 Å by Davis, Bjorkman et al.^16^ This protein is composed of three subdomains: protease (56–116 and 152–591), apical (117–351), and helical (592–750). The apical part, also referred to as the protease-associated domain, is the catalytic domain and contains two β-sheets, one helical turn, and three α-helices (Figure 2).^16^

**Figure 2:**
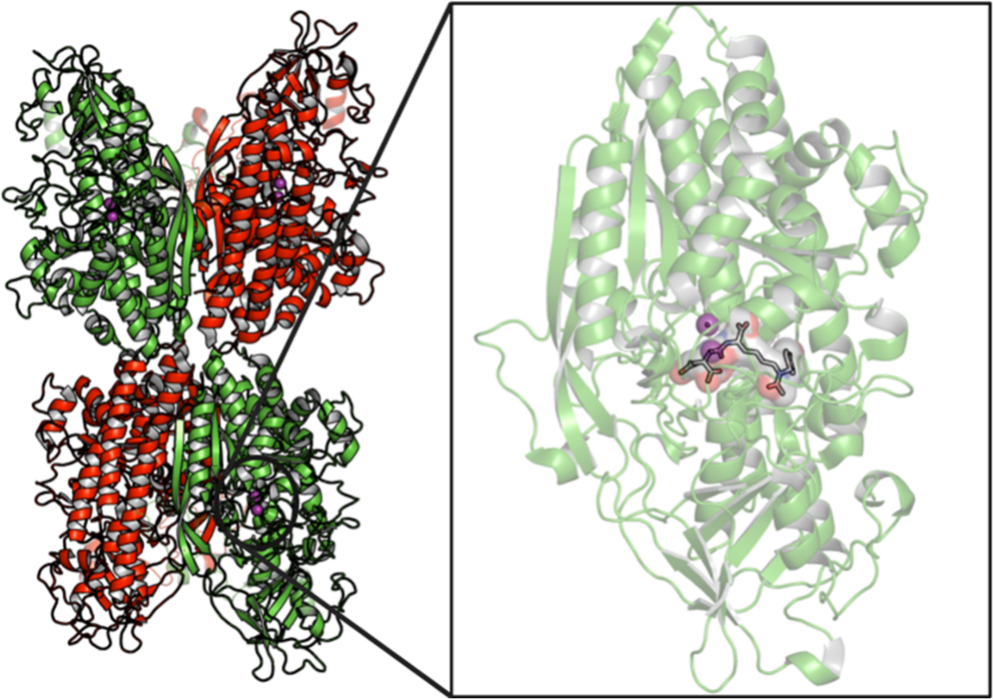
(Left) Structure of the PSMA/GCPII (PDB ID 1Z8L)^16^ enzyme resolved by X-ray crystallography (homodimeric structure and the enzyme active site region). The two parts of each monomer are shown with green and red colors. (Right) The region of the binding area with bound ligand (*S*)-2-(3-((*S*)-1-carboxy-(4-iodobenzamido)­pentyl)­ureido) pentanedioic acid (DCIBzL, **6**, Figure 5)^26^ to the enzyme’s active site area is shown on the right part (protein is shown in light green color and ligand in sticks; the binding area region is shown with van der Waals spheres and the two Zn^2+^ ions are shown in magenta). (Figure 2 was created using the Maestro interface, Schrödinger Release 2021–2: Maestro, Schrödinger, LLC, New York, NY, 2021.).

The active site of PSMA/GCPII (where the substrate binds), located in an apical cavity, includes two Zn^2+^ ions, essential for hydrolytic activity and crucial for binding both the substrate and the inhibitors. The Zn^2+^ ions are both coordinated by two histidine residues (His553 and His377), glutamic acid (Glu425) or aspartic acid (Asp453), and a water-bridged aspartic acid (Asp387). The active site is bordered by two binding pockets, S1′ and S1.^16^ The S1′ pocket is restrictive with regard to available space and is positively charged. Therefore, the S1′ pocket binds with significant selectivity glutamate residues and thus is very sensitive to glutamate-containing peptides.^21,26^ Specifically, Arg210 and Lys699 in S1' interacts with glutamic acid carboxylate groups (or glutamate-like moieties), which are essential for binding interactions with peptides.^21,26,38,39^ Unlike the S1’ pocket, the S1 pocket allows for some binding flexibility but is shallow and is known as arginine patch, which contains Arg463, Arg534, and Arg536 (Figures 2 and 3).^21,26^

**Figure 3:**
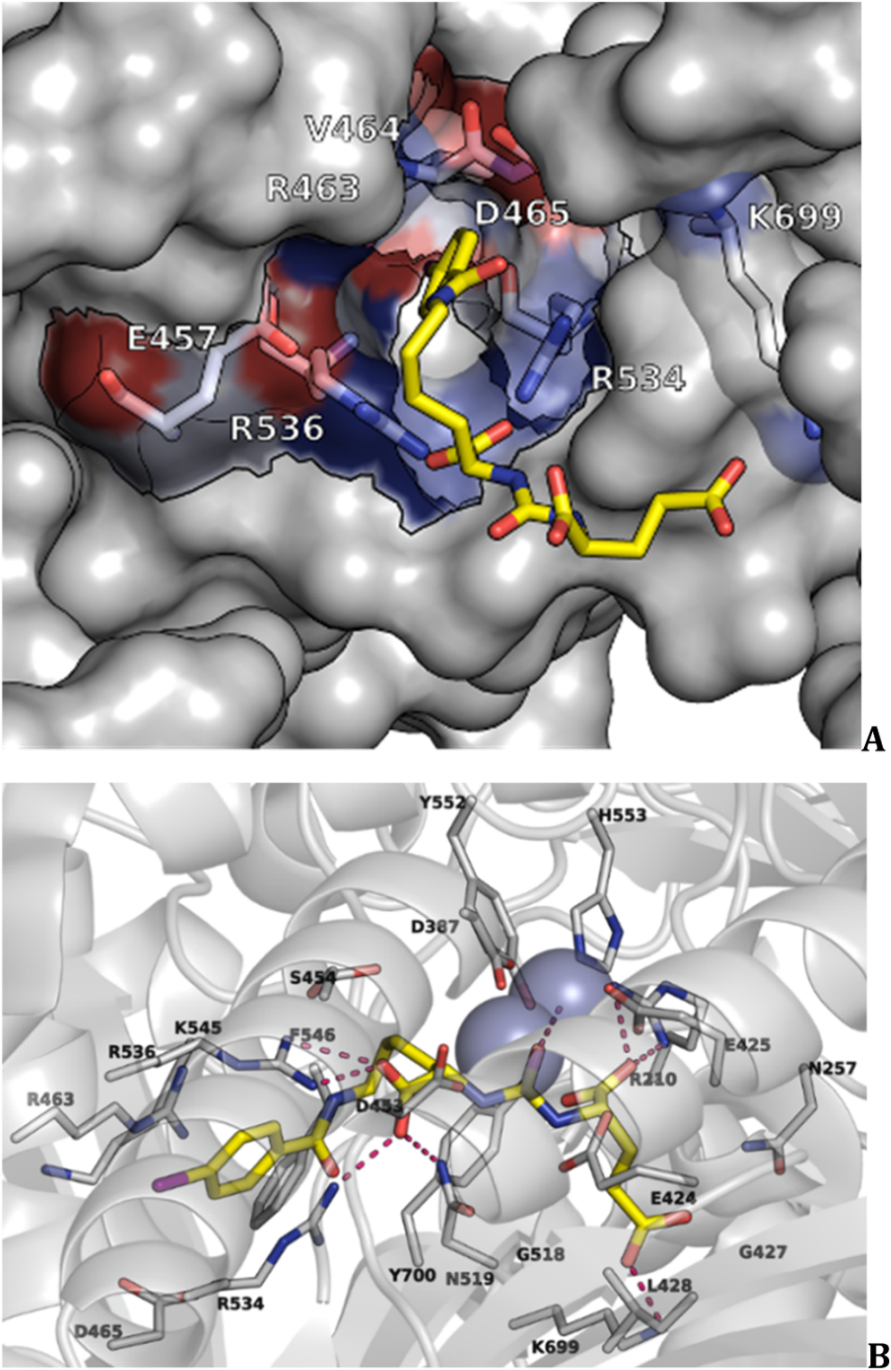
(A), (B) Binding interactions of the Glu-ureido–Lys ligand DCIBzL (6, Figure 5) with active center zinc atoms and the S1, S1′ pharmacophore pockets PSMA/GCPII (PDB ID 3D7H).^26^ In (A) are shown the ureido carbonyl oxygen engaged by the side chains of Tyr552 and His553, a bridging H_2_O and the catalytic Zn^2+^; in S1' the major interactions are the hydrogen bonding interactions of Arg210 and Lys699 side chains with the glutamic acid carboxylate groups while water mediated hydrogen bonding interactions with Asn257 and Tyr700 side chains are also formed; in S1 pocket the arginine patch including Arg463, Arg534, Arg536 and Asn519 binds to the lysine (P1) carboxylate of the ligand. The active-site Zn^2+^ ions are colored blue. Compound DCIBzL bound to the active site is in stick representation (carbons in yellow, oxygen in red, nitrogen in blue, bromine in magenta); hydrogen bonds are shown with red dotted lines. In (B) is also shown the hydrophobic area of the entrance lid which is accessory to the S1 site (bottom to top view). The binding cavity of PSMA/GCPII is shown in semitrans-parent surface representation (gray surface for carbons while oxygen and nitrogen surface are depicted in red and blue). The side chains of amino acids delineating the “accessory hydrophobic pocket” in the entrance lid are shown in stick representation. (Figure 3 was created using Maestro interface, Schrödinger Release 2021-2: Maestro , Schrödinger, LLC, New York, NY, 2021). (**6**, Figure 5).^26^

The distance between the center-of-mass (COM) of the zinc ions and the COM of S1' pocket (arginine patch; Arg463, Arg534, Arg536) or the COM of S1 pocket (Arg210) is ∼ 11-12 Å. The binding ensemble can bind a ligand through a variety of hydrogen bonding and ionic interactions. The S1 pocket also has an accessory S1 site that can bind aryl groups. The Arg536 residue can adopt the two different “binding” and “stacking” conformations. In the “stacking” conformation, the guanidinium group of Arg463 lies between Arg534 and Arg536, and does bind the substrate, but in the “binding” conformation, this residue guanidinium group shifts by 4.5 Å toward the substrate. Upper to the S1 pocket is formed the arene-binding site that comprises Arg463, Arg511, and Trp541, allowing for π-stacking and weak π-cation interactions.. Despite the unknown function, the arene-binding site^27^ has played a role in PSMA/GCPII inhibitor development.^28^ A funnel area is defined between the active site apical cavity and the entrance of the binding area in the water solu-tion region. The distance between the active site apical cavity and the entrance of the funnel ∼ 20 Å length while the funnel width is ∼ 7-8 Å (Figures 2 and 3).

### Pharmacophores Binding to Enzyme’s PSMA/GCPII Active Site Area

A pharmacophore can bind the active center PSMA based on its structural properties and physiological protease-based activity. A wide range of low molecular weight ligand scaffolds^29–31^ have been developed for targeting PSMA/GCPII, which can be “armed” with probes for diagnosis (radionuclides emitting γ radiation or positrons^29,30,32^ or dyes emitting fluorescence^33–35^ ) or toxophores for therapy (radionuclides emitting β or α particles or Auger electrons^29,30,36–39^ or cytotoxic drugs^40–42^ or even cytotoxic proteins^43^).

Several SMDCs^11,44^ have been developed targeting PSMA/GCPII. The first category of ligands for SMDCs is peptides, with sequences either determined by phage-displayed peptide libraries, e.g., SHSFSVGSGDHSPFT (peptide 562, IC_50_ = 661 μM), GRFLTGGTGRLLRIS (peptide 536, IC_50_ = 708 nM),^45^ (WQPDTAHHWATL)_2_KK-biotin (2.2 μmol/L),^46^ GTIQPYPFSWGY (GTI peptide 8.22 μM LNCaP)^47^ or based on the natural PSMA/GCPII substrate poly-Glu chains, e.g., Asp-γGlu-γGlu-Asp-Glu (12ADT-βAsp-Glu-γGlu-γGlu-γGlu IC_50_ = 191 nM, LNCAP cells).^48^

In addition, numerous ligands for SMDCs have been developed, which are peptidomimetic analogs^18,29,49^ of the dipeptide NAAG. These peptidomimetic analogs mimic the transition state of the hydrolysis reaction by the enzyme, while the peptide bond of the substrate is substituted by a hydrolysis-resistant group, such as the phosphorus-containing groups, phosphonate, phosphate, or phosphoramidates introduced by Jackson et al. 1996 ^50^ or the urea group first introduced by Kozikowski et al. in 2001.^51^ The peptidomimetic analogs, which act as inhibitors of the PSMA/GCPII enzyme, can be classified into the following groups based on their chemical structure:

(a) Phosphorus derivatives (including phosphonate, phosphate, and phosphoramidates),^28,50,52–56^ which were the first high-affinity PSMA/GCPII ligands with nanomolar inhibition constants (*K*_i_) but their high polarity and poor pharmacokinetic profile render them unsuitable for clinical applications. Nevertheless, the 2-phosphonomethyl pentanedioic acid (PMPA) synthesized by Jackson and collaborators in 1996 ^50^ showed *K*_i_ ∼ 0.275 nM. This ligand which belongs to the phosphonate chemical class is still the most tightly bound inhibitor and one of the reference inhibitors for blocking PSMA/GCPII in radiolabeled binding experiments when the displacement of radioactive tracers is studied.
(b) Thiol-based ligands^57,58^ were designed to supplant phosphorus-containing molecules, offering improved membrane permeability and higher oral bioavailability. Nevertheless, their low metabolic stability and limited selectivity prevent them from being seriously considered for clinical trials.
(c) Thiourea analogues^59^ were created by replacing the zinc-coordinating urea group with a thiourea group. However, the thiourea analogue (HO-Glu-C­(S)-Glu-OH) demonstrated a binding affinity to PSMA that was 100 times weaker than the urea analogue (OH-Cys-C­(O)-Glu-OH), leading to its discontinuation in further studies.
(d) To address previous compounds limitations, high binding affinity and easily synthesized urea-based PSMA/GCPII ligands have been developed,^29,51,60–66^ as well as carbamate-based ligands.^60^ Between the urea-based ligands, the most common are (S)-Glu-C­(O)-(S)-Lys or EuK (**1**) i.e., PSMA-11 (*K*_i_ = 12.0 nM),^61^ PSMA-617 (*K*_i_ = 2.34 nM),^67^ and (S)-Glu-C­(O)-(S)-Glu or DUPA (**2**) (*K*_i_ = 8.0 nM).^49^

Numerous patents have been filed, between 1/2017 and 7/2020, regarding PSMA-targeting ligands based on the EuK or DUPA structure.^38^ As shown in Figure 4, EuK (**1**) is typically functionalized from Lys, incorporating elements such as a linker, chelator, and dye or toxophore, while DUPA (**2**) is functionalized from one of the Glu moieties.

**Figure 4:**
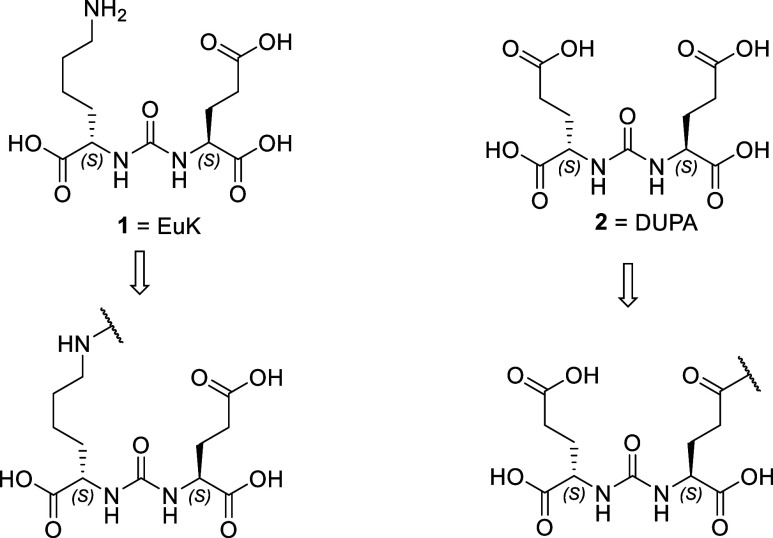
EuK (**1**) and DUPA (**2**) are the two most often used pharmacophores targeting PSMA/GCPII.

Some peptidomimetic ligands occupy only the S1′ binding pocket,^18,21,50,68^ while EuK (**1**),^26,60^ DUPA (**2**)^51^ (Figure 4), and ligands containing an invariant glutamate moiety bind to both binding pockets S1, S1′ and therefore inhibit GCPII.^18,21,29,49,50,68–70^ In these inhibitors the P1′ glutamate carboxylate side chains forms ionic hydrogen bonding interactions in S1′ pocket with Arg210 and Lys699 side chains and the P1 lysine carboxylate side chain with Arg210 and Lys699 within the enzyme’s restrictive S1′ pocket (Figure 3).^21,26^ In EuK and DUPA, the peptide bond is replaced with a urea. The ureido group (−HN–CO–NH−) mimics the planar peptide bond (−CO–NH–, P1′) of the γ-Glu-Glu GCPII substrate (Figure 1), in the complex with the catalytic Zn^2+^.^26^ The interactions within the S1 pocket are principally determined by a network of hydrogen bonds formed between the inhibitor P1 carboxylate group and the side chains of Arg534, Arg536, and Asn519. As mentioned, the S1 pocket is more flexible, and thus several structural modifications in PSMA/GCPII inhibitors have been studied aiming at increasing binding affinity. It has been shown that in EuK^26^ or carbamate analogs,^60^ the affinity is increased with an aromatic substituent connected with Lys residue which binds the hydrophobic pocket accessory to the S1 site (Figure 3).^26^

In another study of EuK-low molecular weight bioconjugates, when the authors replaced Glu in EuK with Asp or 2-aminohexanodioic (l-2-aminoadipic/Aad) or 2-aminoheptanodioic (2-aminopimelic acid/Api), the affinity was reduced detrimentally in the Api analog.^71,72^

## Design of Theranostic PSMA/GCPII Ligands

The general structure of radioligands (**3–11**, Figure 5) contains the following parts: (a) the PSMA/GCPII-targeting pharmacophore (EuK, **3–9** or DUPA, **10**), (b) a part called spacer or linker, connecting the pharmacophore motif with a radiolabeled chelator, and (c) the chelator group, which forms a complex with a radionuclide for diagnosis (SPECT or PET molecular imaging) or endoradiotherapy (α or β particle emitting radionuclides). In some cases of C–H bond radiolabeling with halogens, e.g., [^18^F]­F_2_ (**5**, **7**, **11**), the chelator group was replaced with an aromatic moiety (SNAr, SN_2_ radio-fluorinations)^30^ (the methodology of C–H labeling with [^18^F]F has been recently reviewed by Wright et al.).^73^

**Figure 5:**
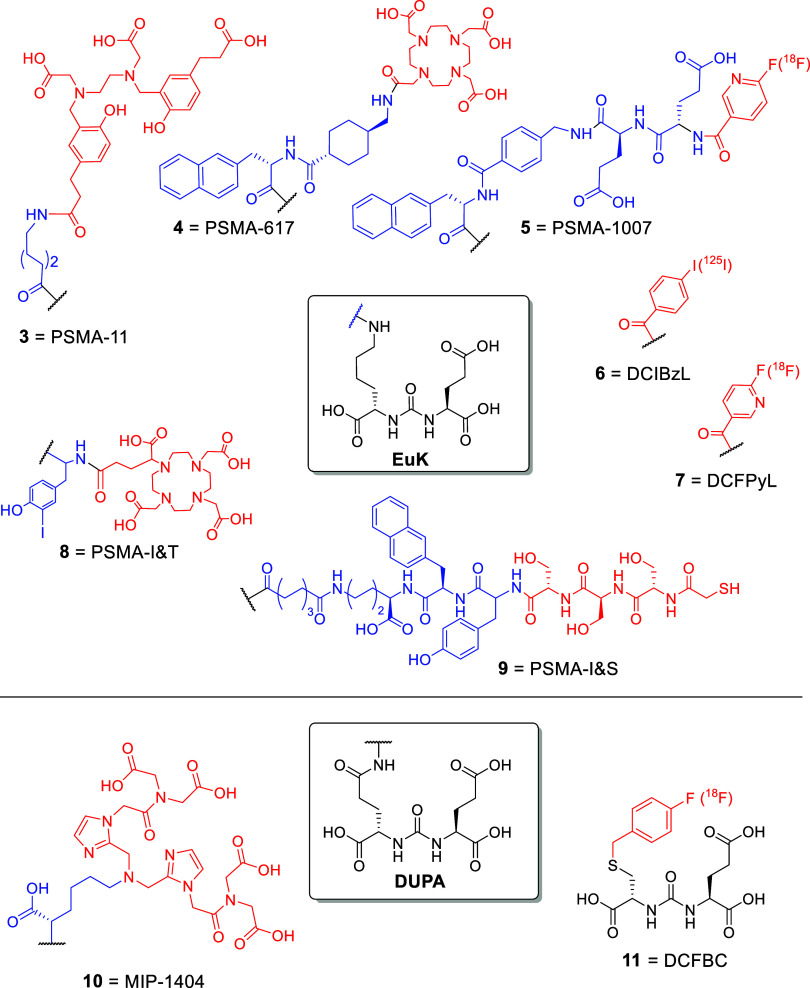
Chemical structures of SMDCs as radioligands targeting PSMA for theranostic applications, containing the peptidomimetic pharmacophores EuK (**3–9**) or DUPA (**10**), or others **11** (black: pharmacophore; blue: linker; red: chelator or aromatic group carrying a radio-halogen).

The linkers in radioligands can vary in length, structure, and lipophilicity. It functions mainly as a mean to increase the distance between the chelator and the pharmacophore and thus to minimize its interference to the binding pocket (e.g.,aminohexanoic acid, PSMA-11 (**4**),^74^ Lys, MIP-1404 (**10**)^75^), while in some cases linkers have also been used as pharmacokinetic modifiers (e.g., 2-naphthyl-l-Ala connected with trans-4-(aminomethyl)­cyclohexanecarboxylic acid, PSMA-617 (**5**),^67^ iodo-d-Tyr-d-Phe-d-Lys-OH, PSMA I&S (**8**),^76^ and PSMA I&T (**7**)^77^).

In some cases of SMDCs, the chelator group has been replaced by a dye (**12**), i.e., IRDye800CW and IRDye800RS,^78–81^ BODIPY,^82^ S0456,^83^ FAM-5,^84^ Cy5,^85^ Cy5.5,^78^ Cy7,^78^ SulfoCy5,^84^ or SulfoCy7,^33,84^ for fluorescent or optical/fluorescence nuclear imaging applications, allowing preoperative prestaging/staging, treatment planning, and surgical (fluorescent) tumor resection possible (**12**, Figure 6).^30,86,87^ The principle of application is the same with the radiometal ligands, but they are safer for the operational personnel, who in this case is using NIR fluorescence operative guidance for precision operation. However, none of these ligands have been approved yet for clinical application.

**Figure 6:**
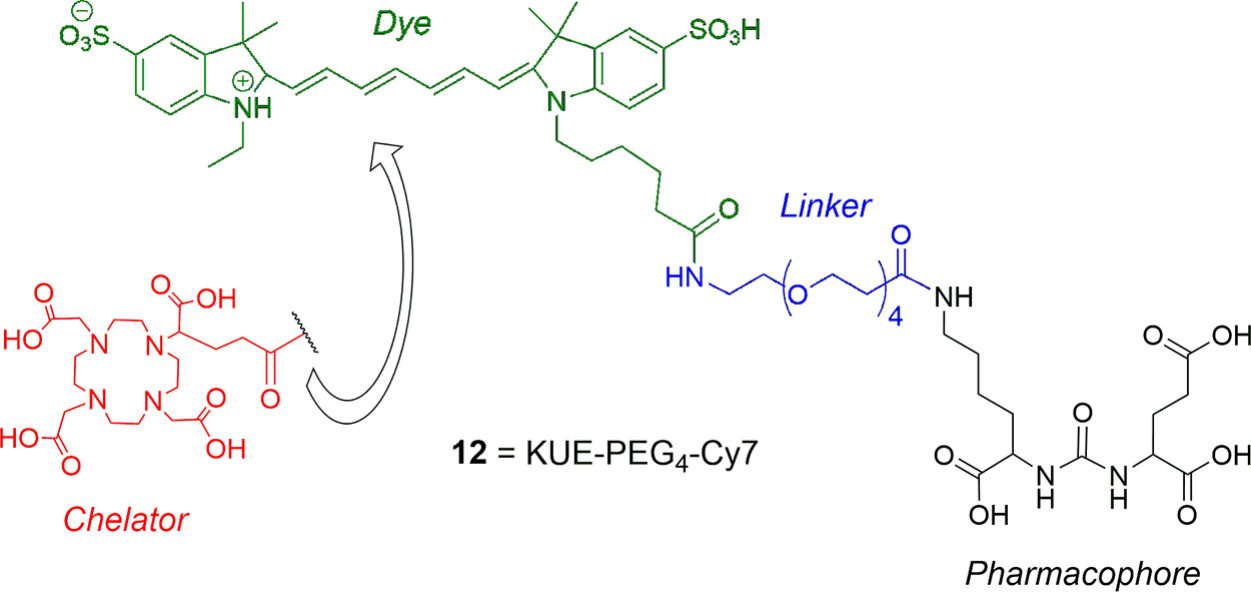
Example of chemical structures of SMDCs (**12**, KUE-PEG_4_-Cy7), where the chelator has been replaced by a fluorescent dye Cy7 (black: PSMA pharmacophore (EuK); blue: linker; red: toxophore/chelator; green: dye).^33^

## LTDs Targeting PSMA/GCPII with Cytotoxic Drugs

### Design Principles of LTDs

The vast development of PSMA-targeting radioligands led to research on PSMA-targeting LTDs, where the chelator was replaced by a cytotoxic drug. LTDs targeting PSMA/GCPII are composed of (a) the targeting pharmacophore, (b) a spacer, a cleavable linker, and (c) a cytotoxic drug (toxophore). Following the conjugate’s cell internalization is the linker’s cleavage. The linker connects the pharmacophore and the cytotoxic drug and can be cleaved either chemically, e.g., through hydrolysis in the acidic medium of endosomes, or by enzymes. The linker cleavage follows the activation of the cytotoxic drug (toxophore),^9–11,44,88,89^ which then is ready to act facilitating apoptosis and cell death. The linker should also be relatively stable during the conjugate’s blood circulation to ensure its arrival at the tumor intact, yet sufficiently labile to enable rapid release of the therapeutic agent.^90^ A limiting factor for targeted drug therapy utilizing receptor-mediated endocytosis is the concentration of the delivered drug, since most pathways deliver only limited quantities of the ligand, reducing thus the efficacy of the used bioconjugate. Therefore, the cytotoxic agent should be of high toxicity for cancer cells.

In addition, the cytotoxic drug should contain one of the chemical groups, −SH, −COOH, −OH, or −NH_2_ to be adapted for intracellular release after the breaking of the covalent bond of the linker.^9–11,44,88,89^ The design of the cleavable linker is of vital importance for the efficacy of these kinds of prodrugs.^10,91,92^ Two basic options are available for selecting the optimal linker for LTDs:

(i) Chemically cleavable acid-labile linkers,^10,91,92^ such as those having (Figure 7): (i-a) a hydrazone bond,^93^ (i-b) an ester, or (i-c) a carbamate bond.^94^ The linkers are stable at pH = 7.4 but can be cleaved in the acidic environment of endosomes (pH = 5.5–6.2) and lysosomes (pH = 4.5–5.0). Additionally, the anaerobic glycolysis (Warburg effect or upregulated glycolysis)^95^ inside the tumor cells produces lactic acid, which causes an acidification of the tumor (pH 6.2–6.8). The cytotoxic drug is delivered often with a free amino or hydroxyl group after linker’s cleavage.^13^

**Figure 7:**
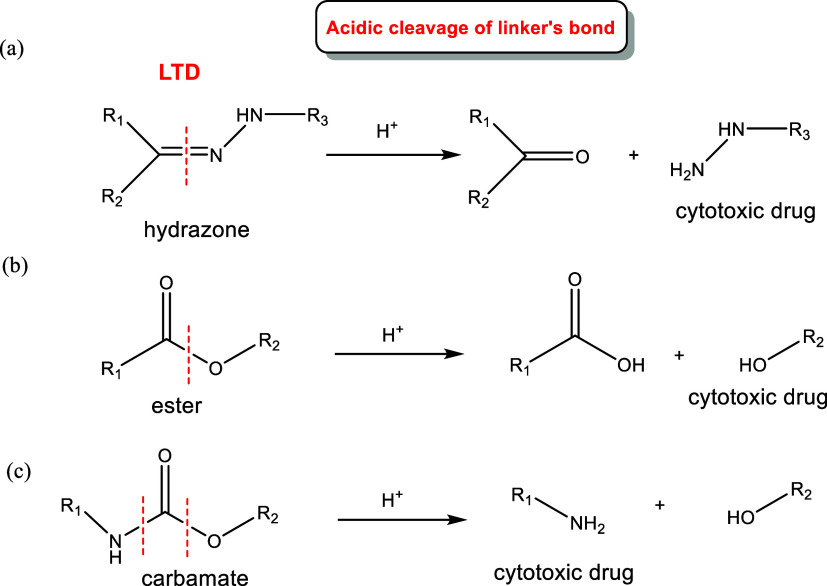
Examples of pH-sensitive bonds hydrazone (a), ester (b), and carbamate bonds (c) in linkers used in LTDs and mechanism of release of the cytotoxic drugs in the cells.

(ii) Enzymatically cleavable linkers^10,92,96^ (Figure 8).

**Figure 8:**
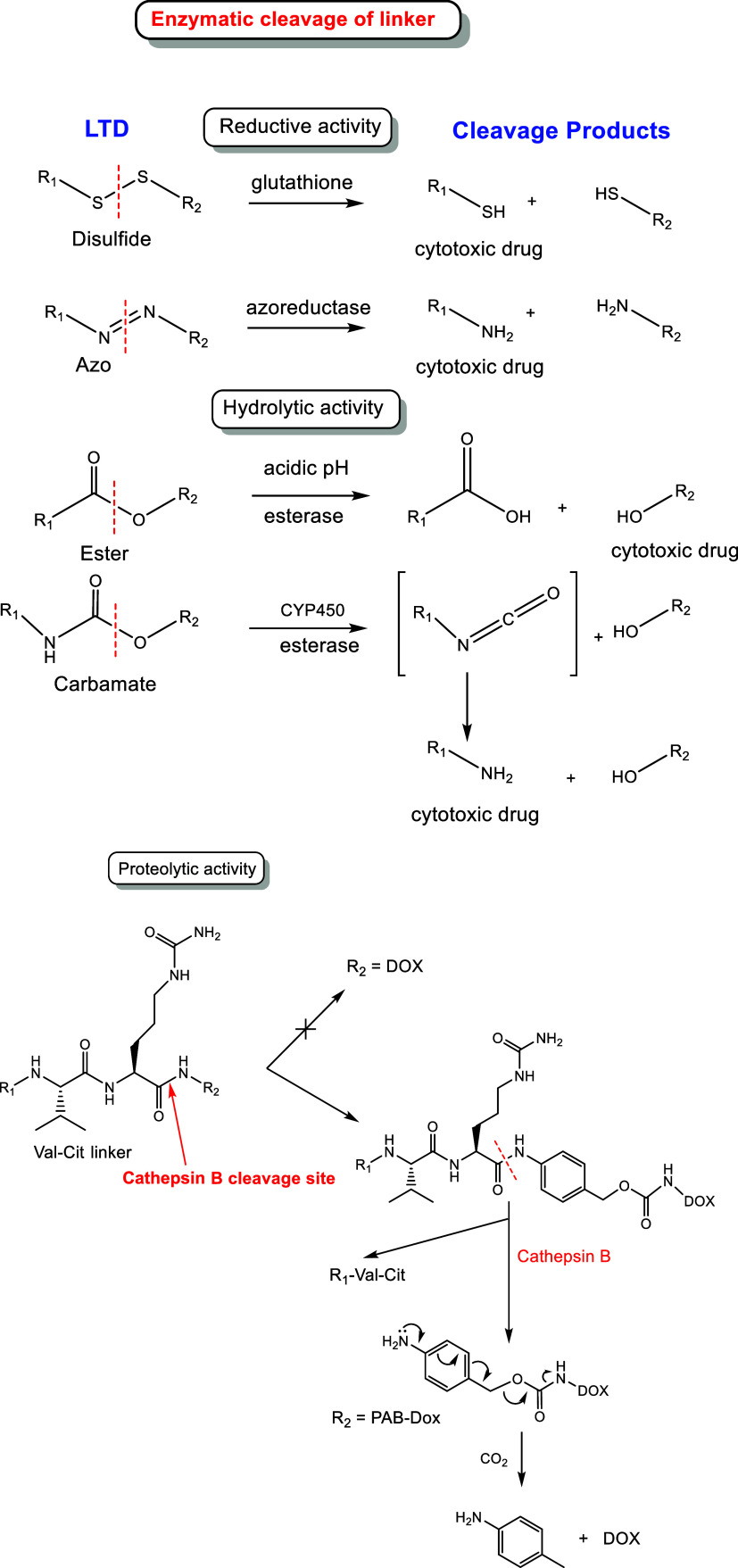
Examples of enzymatically cleaved linkers in LTDs and mechanisms of cytotoxic drug release in cancer cells, i.e., reduction of disulfide and azo bonds; hydrolysis of ester, carbamate, or amide bonds (VCitP) in the low lysosomal pH (pH = 4.5–5.0), by esterases, cytochrome P450 (CYP450), or proteolytic activity by cathepsin protease B, respectively.

(ii-a) Linkers with a disulfide bridge (S-S).^10,97^ This can be reduced by glutathione reductase (GR)/glutathione-disulfide reductase (GSR), producing two thiol-bearing compounds.

(ii-b) Azobenzene linker.^98^ Azoreductases reductively cleave azo linkages using NAD­(P)H as an electron donor.

(ii-c) Linkers with ester or carbamate^99,100^ bonds. A category was also included in the previous classification of chemically cleaved linkers. These two categories offer selective cleavage in cancer cells by hydrolytic enzymes, e.g., carboxylesterases or carboxypeptidases, which deliver the cytotoxic drug with a free hydroxyl or amino group, correspondingly.

(ii-d) A peptide sequence which releases the drug after proteolysis by proteases,^101^ e.g., cysteine cathepsins. In general, cysteine cathepsins are highly upregulated in a wide variety of cancers; they can be found on cell membranes, or secreted and localized in endosomal or lysosomal vesicles, which suggest a change in their enzymatic substrates and functions depending on their location.^102,103^ Thus, cathepsin B, which is located in the lysosomes, is an important target for cancer^10,104^ and is overexpressed in invasive and metastatic cancer phenotypes.^102,103^ In some cases, attaching a drug with a peptide linker may be sufficient for cytotoxic payload delivery. However, the drug-peptide linker chemical bond may suffer from steric hindrance during the proteolytic cleavage,^105^ as in the reference cases of Z–Phe–Lys–DOX and Z–Val–Cit–DOX (DOX = Doxorubicin) prodrugs tested with cathepsin B.^106,107^ To overcome this drawback, the *p*-aminobenzyloxycarbonyl (PABC) group, that belongs to the self-immolative spacers,^105,108^ is frequently utilized, e.g., the linker VCitP (valine–citrulline-*p*-aminobenzyloxycarbonyl/Val-Cit-PAB/VCitP). VCitP was attached to DOX producing Z–VCitP-DOX, which can release DOX under the enzymatic action of Cathepsin B (Figure 8).^105,108^ The VCitP linker has often been used with antibody drug conjugates (ADCs)^10^ because it is cleaved selectively from cathepsin B over other cysteine cathepsins.^34,35,109^

The VCitP linker combined with a carbamate group delivers the drug with a free amino group through decarboxylation (Figure 8). Therefore, in the case of self-immolative spacers,^105,108^ the spacer in the linker is needed for the activation mechanism.

The role of the linker is not limited to facilitating the release of the cytotoxic drug; it can also affect (1) the binding of the LTD pharmacophore, i.e., increase its affinity (by binding to the accessory hydrophobic S1 pocket or the arene site^63^), (2) the stability of LTD, i.e., replacing the VCit linker in VCitP with the tripeptide glutamic acid–valine–citrulline (EVCit)^110^ improved the stability in mouse serum, and (3) the pharmacokinetics of LTD, i.e., extension of blood circulation time due to its binding to trasthyretin.^111^ Numerous such studies regarding the role of the linker in pharmacokinetics have been described in radiopharmaceuticals.^112,113^

### LTDs with Enzymatically Cleavable Linkers

#### LTDs Containing the Poly-Glu Pharmacophore

In 2004, Denmeade et al.^114^ synthesized and tested several methotrexate (MTX)-based peptide LTDs (**13**, Figure 9) to identify PSMA selective substrates, which would be resistant to hydrolysis (nonspecific) in human and mouse plasma, but susceptible to PSMA enzymatic action and drug activation. For this in the Poly-γGlu-MTX analogues synthesized, the α-linked Glu residues were replaced by γ-linked Glu residues, i.e., LTD **13**. The poly-γGlu peptides, containing α- and γ-linked Glu, are substrates of PSMA subjected to enzymatic hydrolysis in the plasma membrane of LNCaP cells.^114^

**Figure 9:**
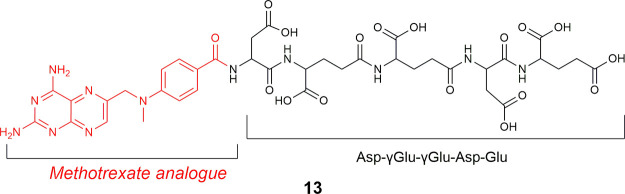
Chemical structure of LTD **13** targeting PSMA/GCPII. In LTD **13**, 4-(*N*-((2,4-diaminopteridin-6-yl)­methyl)­lamino)­benzoic acid/4-amino-N10-methylpteroic acid (APA), an analogue of MTX, is coupled with PSMA/GCPII-targeting oligopeptide Asp-γGlu-γGlu-Asp-Glu (black: PSMA pharmacophore; red: cytotoxic drug (MTX analogue).

Since a poly-Glu is a known substrate for PSMA, the MTX-based peptide bioconjugates were evaluated for their *in vitro* stability and selective toxicity using PSMA-expressing, PSMA­(+) LNCaP PCa cells and non-PSMA expressing, PSMA(−) TSU, human bladder cancer cells. The bioconjugates containing longer chains with γ-linked Glu residues were more efficiently hydrolyzed in PSMA­(+) cells but were unstable in plasma. However, the bioconjugate analogs containing both α- and γ-linked Glu residues were less efficiently hydrolyzed in the PSMA­(+) cells but were stable in plasma. The γ-linked Glu bioconjugates showed potent cytotoxicity (1–10 μM), but they were not significantly hydrolyzed to deliver MTX in PSMA(−) TSU cells. It was suggested that their transportation into cells might be mediated by folate receptors (FR-α, FR-β), which specifically recognize the pteridine moiety of folate and antifolates.^114,115^ Owing to this observation, it was suggested that future LTDs targeting PSMA should not contain MTX or other antifolates.

The same group developed in 2001 ^116^ a PSA-targeting LTD based on the cytotoxic drug TG (**14**) ( Figure 10). TG (**14**) is a natural product isolated from the Mediterranean plant *Thapsia garganica* and an inhibitor of sarcoplasmic/endoplasmic reticulum calcium ATPase (SERCA). It has a proliferation-independent toxicity for cancer cells and high toxicity *in vivo* for normal cells.^117^ Even low nanomolar doses of TG induce apoptosis in both proliferating and nonproliferating cells. To target the powerful cytotoxicity of TG exclusively to tumor cells, a protease-activated prodrug was developed in which TG (**14**) was attached to a PSA (prostate-specific antigen) protease-specific peptide pharmacophore after esterification in the O-8 position with ω-amino acids [HOOC­(CH_2_)*_n_* NH_2_, *n* = 5–7, 10, 11]. It was found that the activity of these aminoacyl prodrugs increased analogously to their length, thus 12ADT (**15**) was selected, while L12ADT (**16**) was equipotent to TG (**14**).^116^ The L12ADT (**16**) was coupled to various PSA pharmacophores, e.g., QLKSSH-Mu (Mu = 4-morpholine-carbonyl amino-terminal protecting group), which produced a soluble, cell-impermeant latent LTD **17** (Figure 10).^116^ PSA is a proteolytic enzyme located in the extracellular fluid of prostatic cancer cells. It has chymotrypsin-like activity and it preferably cleaves sites between Glu and Ser or Leu.^116,118–120^ **17** was potent against PCa since it was specifically activated extracellularly by PSA at metastatic PCa sites, releasing the cytotoxic L12ADT (**16**).^118^ It is also worth noting that the same group developed in 2007 ^119^ a (*N*-(2-hydroxypropyl)­methacrylamide) HPMA-based polymer covalently linked to the PSMA-activated peptide SSKYQL coupled with 12ADT (**15**).^119^ In animal studies,^118^ the HPMA-based polymer-SSKYQL-TG did not induce apoptosis of PCa cells *in vitro*, while the activated L12ADT (**16**) was released from LTD (**17**) and accumulated in the tumor tissues.

**Figure 10:**
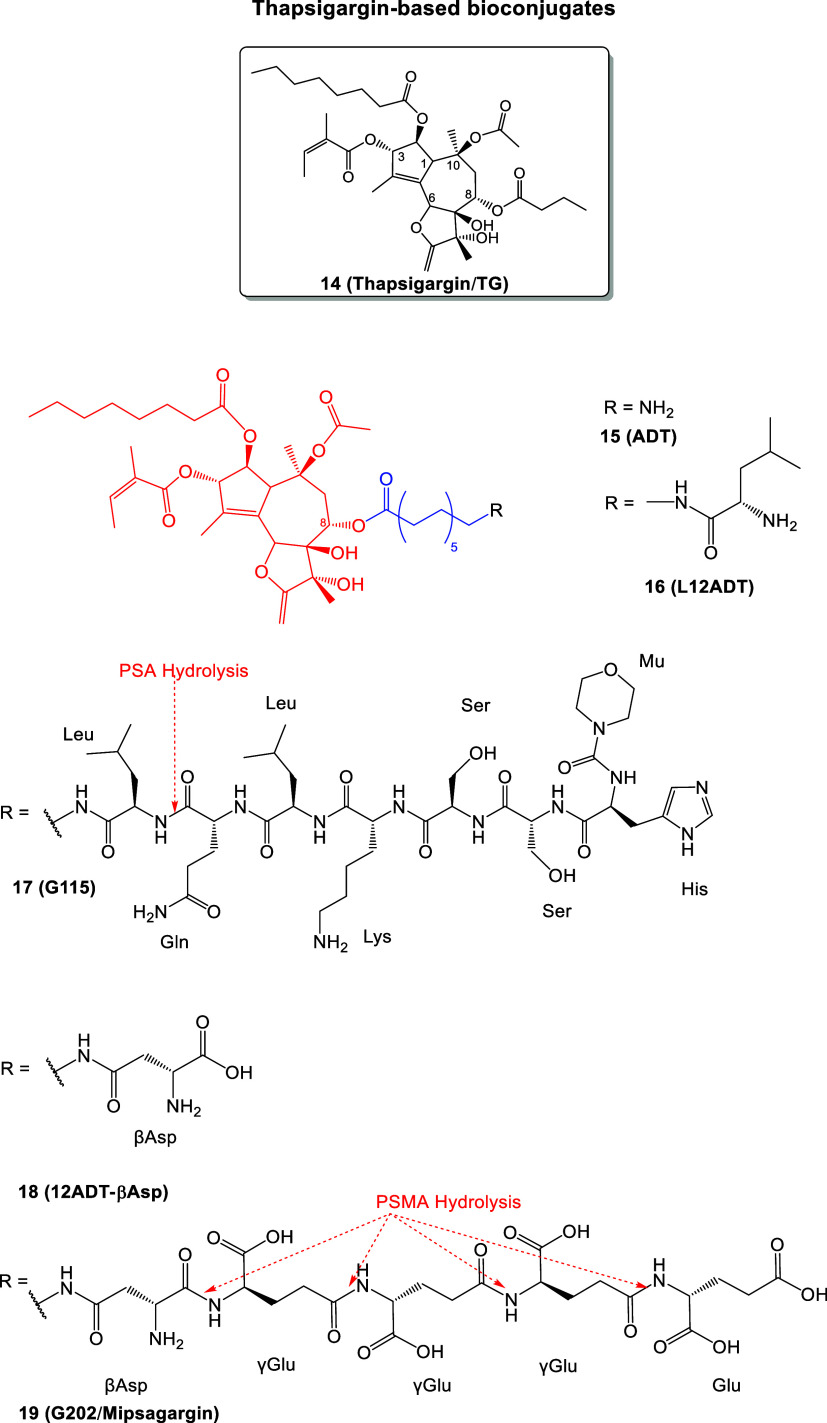
Chemical structures of TG (**14**) and bioconjugates **15–19** targeting SERCA pump through releasing cytotoxic prodrugs of TG (**14**) (black: PSA and PSMA pharmacophores; blue: linker; red: Thapsigargin moiety).

The same group developed in 2012 ^48^ the LTD Mipsagargin/G202 (**19**) (Figure 10) using the PSMA-specific pharmacophore βAsp-(γGlu)_3_-Glu-OH, instead of QLKSSH-Mu.^48^ The release of the cytotoxic 12ADT-βAsp (**18**) by the PSMA/GCPII protease would occur extracellularly. The extracellular activation of G202/Mipsagargin (**19**) could also overcome the problem οf heterogeneity in PSMA/GCPII expression because in this way both PSMA­(+) and PSMA(−) cells would be killed within the peritumoral space.^48^

Regarding the synthesis of G202/Mipsagargin (**19**), solid-phase peptide synthesis (SPPS) protocols using a 2-chlorotrityl chloride resin in combination with solution-phase conjugation were applied.^48^ Similar synthetic protocols were later applied in other bioconjugates of TG (**14**).^120^

In LTD **19**, the poly-Glu peptides act as a substrate for PSMA, while 12ADT-βAsp (**18**) is bound to the sarcoendoplasmic reticulum calcium transport ATPase (SERCA) pump, which transports Ca^2+^ ions from the cytoplasm into the sarcoendoplasmic reticulum.^48^ The functions of this enzyme are important for muscle physiology (cardiac/skeletal muscle contraction), while it also plays a critical role in the control of calcium-dependent cell activation, growth, and survival.^121,122^ Its overall structure consists of three cytoplasmic domains and 10 transmembrane helices. Structural studies of the SERCA pump with 12ADT-βAsp revealed the binding of the pharmacophore (12ADT) mainly to the transmembrane domain with some extensions into the space between the a-helices that make up the transmembrane lipid domain.^48^

In particular, the X-ray crystallographic studies of 12ADT-βAsp (**18**) bound to SERCA pump^48^ revealed that the α-NH_2_ group of the Asp moiety is hydrogen bonded to Gln250 and possibly also with the phosphatidylethanolamine (PE) in cell membranes. In particular, the βAsp moiety occupied a similar binding site^48^ in the SERCA pump with cyclopiazonic acid, a known inhibitor of the SERCA pump.^48^ Although cyclopiazonic acid is bound to a different site than TG (**14**), the authors observed overlapping between the binding sites^48^ of the βAsp linker and cyclopiazonic acid. This was also confirmed by intrinsic fluorescent studies showing the competition of 12ADT-βAsp (**18**) with cyclopiazonic acid for binding to the SERCA pump.^48^

X-ray crystallography of PSMA-ligand complexes (PDB ID 3BI0)^21^ revealed an ∼20 Å deep funnel connecting the water-exposed surface of PSMA (the entrance of the funnel) with COM of the two Zn2+ in the enzyme’s active site, which hydrolyses poly-γ-glutamate chains to glutamate residues.^19,21,26^ Computational studies by Denmeade et al. regarding the PSMA-ligand binding suggested that it could fit LTDs of 12ADT linked to glutamate residues.^48^ More specifically, the 12-carbon side chain of the linker in 12ADT (**15**) had adequate length to keep the sizable toxophore TG (**14**) outside the funnel. This hypothesis corresponded with previous computational modeling work with PSMA -targeting peptide linkers of variable length.^46^ In addition, the five acidic amino acid oligopeptide part βAsp-γGlu-γGlu-γGlu-Glu in 12ADT/G202/Mipsagargin (**19**) had (a) the appropriate length for being completely hydrolyzed by PSMA/GCPII^114^ and (b) a negative charge which enhanced the solubility of lipophilic 12ADT, while preventing the nonhydrolyzed prodrug 12ADT-βAsp (**18**) from penetrating the plasma membrane of normal cells.^48^ A model that we generated with docking calculations is shown in Figure 11.

**Figure 11:**
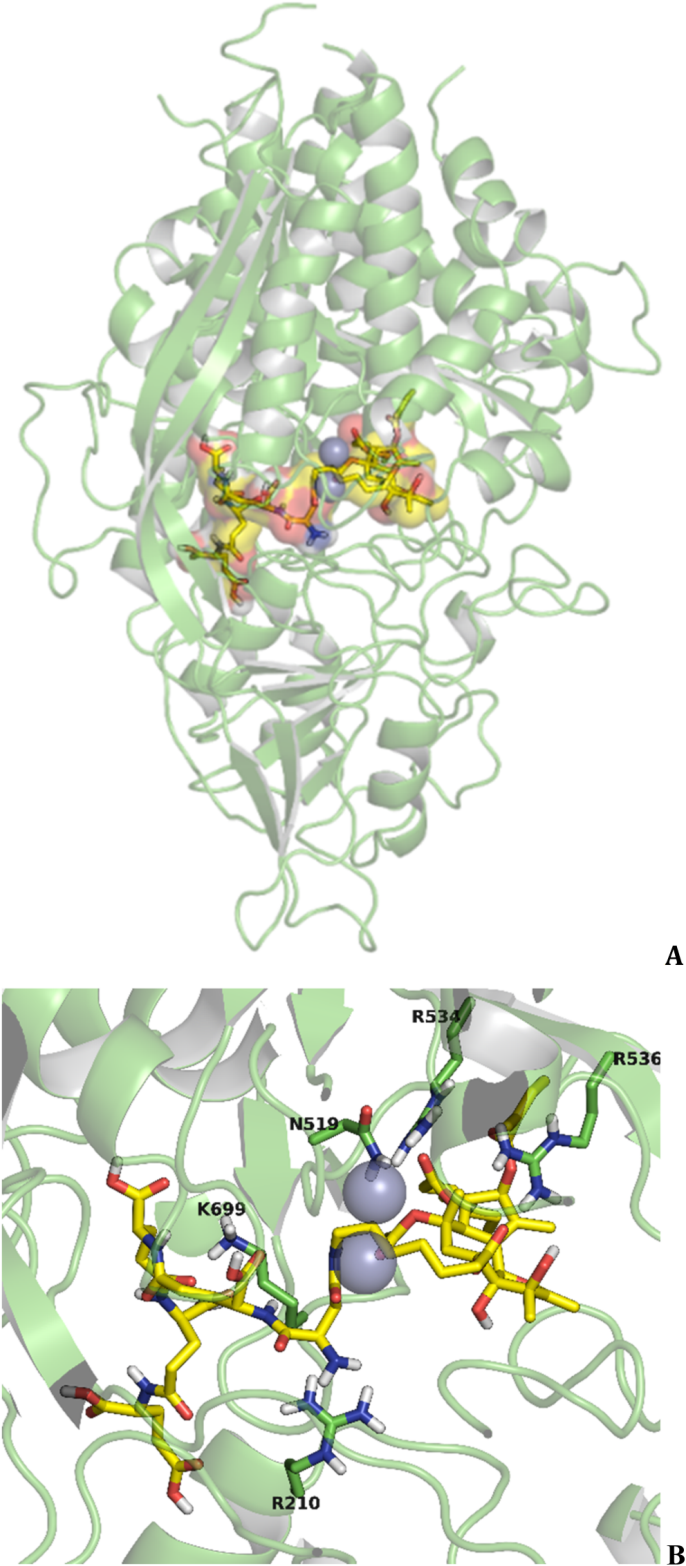
(A) Computational model of the G202/Mipsagargin (**19**)–PSMA complex generated with molecular docking calculations using Glide program and GlideXP scoring function,^123,124^ based on X-ray structure of DCIBzL in complex with GCPII (PDB ID 3D7H^21^) using Maestro interface (Schrödinger Release 2021–2: Maestro, Schrödinger, LLC, New York, NY, 2021). The calculations showed that G202/Mipsagargin (**19**) binds the PSMA/GCPII funnel, with no unfavorable steric interactions. The conjugate βAsp-γGlu-γGlu-12ADT fills the tunnel with TG (**14**) located outside the channel; the protein is depicted in a green cartoon, and the ligand is shown in sticks (ligand: carbon atoms—yellow, oxygen—red, nitrogen atoms—blue color, and polar hydrogens are depicted; protein: green cartoon, binding area: van der Waals spheres, and Zn^2+^ ions—gray color). (B) Zoom in of the binding area. It is shown that the γGlu-Glu terminal fragment of the pentapeptide in LTD G202/Mipsagargin (**19**) binds the S1 and S1′ regions of the binding pocket.

The LTD G202/Mipsagargin (**19**) along with variants of the pentapeptide PSMA/GCPII pharmacophore Asp-(γGlu)_3_-Glu (achieved by altering amino acids in the first and last positions) were also compared.^48^ The results showed that (a) the 12ADT-βAsp-Glu-γGlu-γGlu-Asp analogue was stable to hydrolysis in human plasma but also in the presence of PSMA. (b) The 12ADT-Glu-γGlu-γGlu-γGlu-γGlu analogue was stable to hydrolysis in human plasma, but it was hydrolyzed from PSMA to the dipeptide analogue 12ADT-Glu-γGlu. (c) The βAsp-Glu-γGlu-γGlu-Glu-12ADT analogue was rapidly hydrolyzed to 12ADT-βAsp-Glu. (d) G202/Mipsagargin (**19**) was completely stable to hydrolysis in human plasma. **19** was not hydrolyzed by a variety of other proteases tested, while being specifically hydrolyzed by PSMA/GCPII.^48^

G202/Mipsagargin (**19**) displayed selective toxicity to PSMA­(+) cells, such as LNCaP cells, with an IC_50_ that is 57 times lower than that in the PSMA(−) TSU cells. *In vivo*, G202/Mipsagargin (**19**) showed significant tumor regression in two mouse xenograft models of human PCa and in one model of human breast cancer. Specifically, a single 3-day course/30 days of G202/Mipsagargin (**19**) produced ∼50% regression of LNCaP tumor xenografts and significant antitumor effects in two other PCa xenograft models (MDA-PCa2b and CWR22R-H), in the breast cancer model MCF-7, in renal cell carcinoma (SN12C), and in human bladder cancer (TSU). Also, the effectiveness of LTD **19** was far better than docetaxel (a standard care protocol), while its toxicity was much lower when the maximum tolerable dose was used.^48^ The *in vivo* pharmacokinetic (PK) studies, using BALB/c mice, demonstrated that G202/Mipsagargin (**19**) had a half-life of 4.9 h post administration (single dose = 67 mg/kg), while after 5 days (single dose, *D* = 56 mg/kg), significant accumulation of both the bioconjugate 12ADTβAsp (**18**) and **18**-γGlu was observed in the CWR22R-H tumor tissue compared with the normal kidney, skeletal muscle, and brain.

Based on these preclinical data, 6 clinical studies have been initiated so far, 5 of the 6 have been completed, while 1 has been withdrawn.^40^ Among the 5 completed, the detailed results have been published only for 2 clinical trials, while for the rest of the trials (phase II), only limited data are available so far. Between 2010 and 2013, a phase I (dose-escalation) clinical trial was conducted with G202/Mipsagargin (**19**) in patients with advanced solid tumors^40^ (days 1, 2, and 3/28-day cycles, IV *D* = 1.2–88 mg/m^2^, 44 patients), to determine maximally tolerable dose (MTD). Regarding the safety of (**19**), one case of dose-limiting toxicity (Grade 3 rash) was observed in the dose-escalation portion of the study. At 88 mg/m^2^, observations of grade 2 infusion-related reaction (2 patients) and grade 2 creatinine elevation (1 patient) were observed, which resulted to RP2D = 66.888 mg/m^2^. The most common treatment-related adverse events were fatigue, rash, nausea, pyrexia, and infusion-related reaction. Two patients developed treatment-related grade 3 acute renal failure that was reversible during the treatment-free portion of the cycle. Overall G202/Mipsagargin (**19**) demonstrated an acceptable tolerability and favorable pharmacokinetic profile in patients with solid tumors and thus moved on in several phase II clinical studies.

In an open-label Phase II study, (**19**) was administered IV (days 1, 2, and 3/40 mg/m^2^ or day 1, 40 mg/m^2^, days 2, 3, 66.8 mg/m^2^/28-day cycle) to patients with hepatocellular carcinoma (HCC) who progressed on or to patients after treatment with sorafenib or to patients intolerant to sorafenib drug (Sorafenib/Nexavar, Bayer Healthcare, Whippany, NJ, USA); sorafenib is a protein kinase inhibitor with activity against many protein kinases, e.g., vascular endothelial growth factor receptor (VEGFR) and platelet-derived growth factor receptor (PDGFR) tyrosine kinases and rapidly accelerated fibrosarcoma (RAF) serine/threonine-specific protein kinases. The study aimed in determining the time to disease progression (primary goal), response rate, progression-free survival, overall survival, and safety.^41^ Blood flow metrics in hepatic lesions were evaluated using dynamic contrast-enhanced magnetic resonance imaging (DCE-MRI). Among the 25 treated patients, 19 were evaluable for efficacy. None had a complete or partial response, and none had an objective response. Twelve out of 25 (63.2%) achieved the best response of stable disease, while 12 out of 25 showed radiologic progression. Seven out of 25 (36.8%) were censored. The median time to disease progression was 134 days; the median progression-free survival was 129.0 days, while the median overall survival was 205 days. Disease status was assessed radiologically after each second cycle of treatment. Of five patients with DCE-MRI data for 11 HCC lesions, all demonstrated a reduced volume transfer coefficient, *K*_trans_ (mean, 52%) (one of the most widely recommended end points to evaluate antiangiogenic therapy). The most common treatment-emergent AEs were grades 1 and 2 and consisted of increased blood creatinine (68.0%), fatigue (56.0%), and nausea (44.0%).^41^ G202/Mipsagargin (**19**) was relatively well tolerated and resulted in disease stabilization in patients with advanced HCC, who had progressed on prior treatment with sorafenib, while significant antiangiogenic effects were observed in hepatic lesions.

To overcome the challenge of blood–brain barrier penetration of TG (**14**) or other cytotoxic drugs in patients with recurrent/progressive glioblastoma (GBM), the soluble LTD G202/Mipsagargin (**19**) prodrug was administered.^125^ This was an open-label, phase II trial regarding the application of G202/Mipsagargin (**19**) in adult patients with GBM, following 1–4 prior therapies with an actual goal of 34% efficacy evaluable. G202/Mipsagargin (**19**) was administered IV in two dosing regimens, like the previous Phase II trial. However, due to better tolerability, in this patient population, only the lower dosing regimen was selected for further evaluation. Based on the initial published results of the 13 patients who have been enrolled, 9 were evaluable for efficacy and 2 (22%) have experienced disease stabilization. AEs noted were mostly grade 1 or 2 and were attributed to G202/Mipsagargin (**19**) administration, e.g., reversible creatinine elevation, fatigue, rash, nausea/vomiting, headache, infusion-related reaction, upper abdominal pain, GERD, fever, itching, and intermittent body aches^125^ (G1 = mild, G2 = moderate, G3 = severe, G4 = life threatening, and G5 = death).

#### LTDs Containing GSR Cleavable Disulfide Bond-Based Linkers

In these cases of LTDs, the toxophore is connected to DUPA or EuK via a disulfide bond. The reduction of the S-S bond by glutathione-disulfide reductase (GSR) (Figure 8) within the endosomes releases the toxophore inside the cancer cells. In 2009, Kularatne et al. developed a PSMA-targeting bioconjugate for the diagnostic imaging of PCa (**25**), (DUPA-Aoc-(Phe)_2_-Dap-Asp-Cys, **A**_**0**_) (Aoc = aminooctanoic acid, Dap = 2,3-diamino-propanoic acid)^126^ (Figure 12). The last three parts (Dap-Asp-Cys) formed the chelator group (N_3_S) for the complexation of ^99m^Tc (**25**). The analogues of **25** were also linked with a dye (DUPA-fluorescein and DUPA-rhodamine B).^127^ The group also developed a nonradiolabeled bioconjugate for therapy (**28**) in which the vinca alkaloid tubulysin B (Tub) (in its hydrazide form, Y-R_1_, 20) was connected to DUPA through a linker similar to the one used in the structure of the diagnostic **25** (Aoc-(Phe)_2_-Cys) with some alterations (γGlu-Asp-Phe). Specifically the Aoc and one of the Phe were replaced by more hydrophilic groups to counterbalance the hydrophobicity of tubulysin (A_3_, Figure 12).^127^ Tub, cytotoxic activity relates to its ability to interfere with microtubule dynamics and inhibit tubulin polymerization. Tubulysins are potent molecules against various cancer cells, with IC_50_ values in the picomolar range (Tub, IC_50_ = 0.091–2.3 nM). However, Tubulysins display a limited application in therapy due to their severe toxicity and small therapeutic window, making them unsuitable for clinical use.^128^ The linker length and structure (with the inclusion of Aoc to avoid steric overlap within the narrow regions at the base of the tunnel and (Phe)_2_ to maximize the ligand interaction with hydrophobic pockets near the mouth of the PSMA tunnel) were optimized in the previous study of Kularatne et al. regarding the radiotracers.^126^ DUPA-SS-TubH III­(**28**) effectively killed PSMA­(+) LNCaP cells in culture (IC_50_ = 3 nM) and eliminated established tumor xenografts in mice.^127^ The new drug **28** presented a dramatic increase in its therapeutic index (TI), while maintaining its effectiveness.^127^ Such improvements in TI were also observed for the folate bioconjugates of Tub,^129^ strengthening the case for future development of LTD bioconjugates.

**Figure 12:**
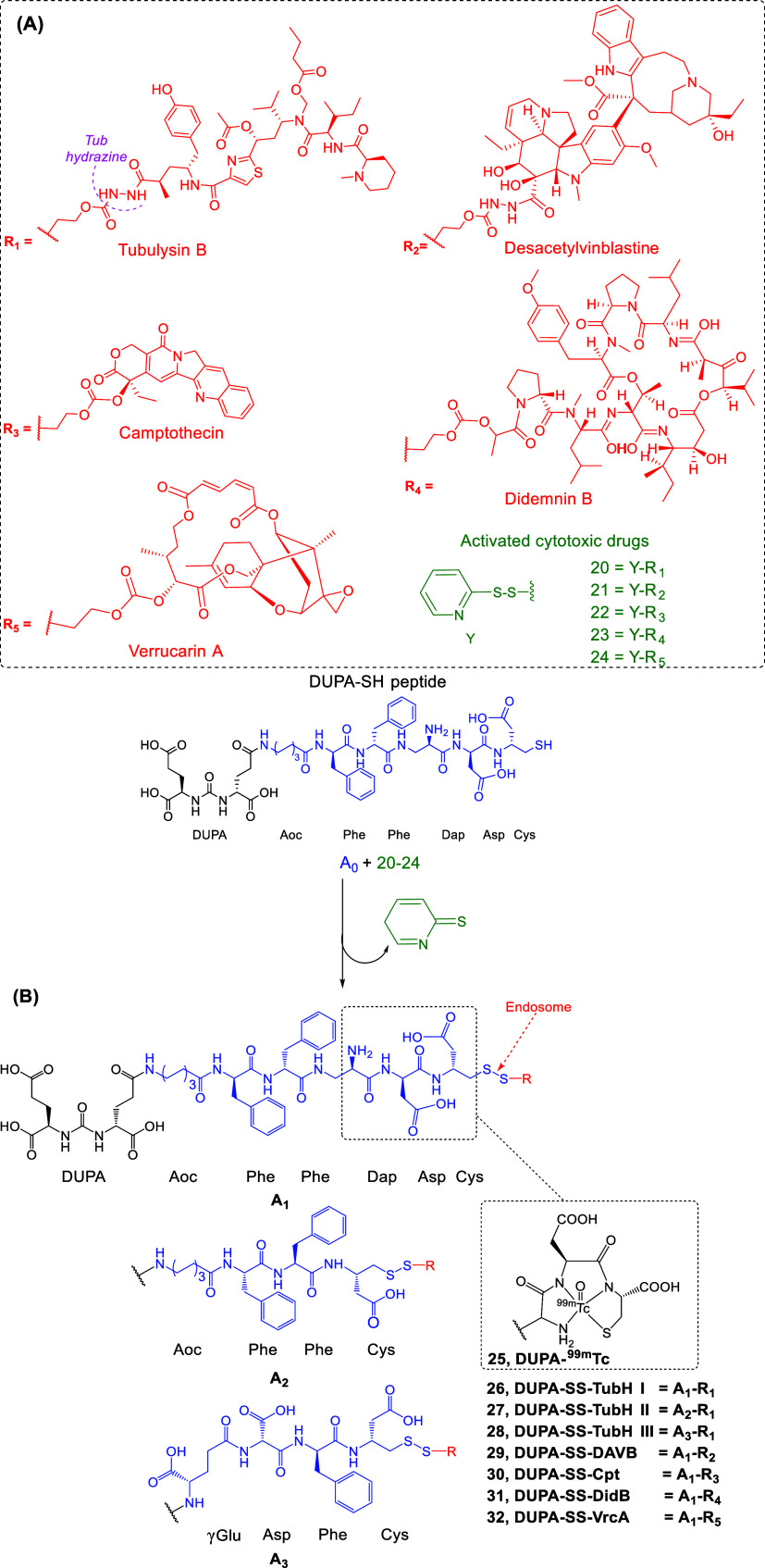
(A) Chemically activated forms of the cytotoxic drugs (Cpt: Y-R_3_ **22**, DidB: Y-R_4_ **23**, VrcA: Y-R_5_ **25**, TubH: Y-R_1_ **20**, DAVBH:Y-R_2_ **21**) were connected through a carbonate ester bridge to a 2-(ethyldisulfanyl)­pyridinyl group (Y) and then used for coupling with DUPA-SH peptide shown at the bottom part of the panel (A_1–3_).^90^ (B) Structures of the PSMA/GCPII-targeting DUPA-^99m^Tc diagnostic **25**, and the disulfide-bridged self-immolative LTDs **26–32**; A_1_ = Aoc-Phe_2_-DAP-Asp-Cys-S-S in **26**, **29–32**, A_2_ = DUPA-Aoc-Phe_2_-Cys-S-S in **27**, A_3_ = γGlu-Asp-Phe-Cys-S-S in **28** (black: PSMA pharmacophore; blue: linker; red: cytotoxic drug) (Aoc = aminooctanoic acid, Dap = 2,3-diamino-propanoic acid).

Based on those promising results, the same group^90^ synthesized a series of DUPA-based prodrugs (Figure 12, **26–32**) using (i) Tub (R_1_) and desacetylvinblastine (DAVB) (R_2_), both tubulin inhibitors, (ii) antimitotic drugs such as camptothecin (Cpt) (R_3_) (topoisomerase I inhibitor), (iii) didemnin B (DidB) (R_4_), a cyclic depsipeptide, which activates caspase (protease enzymes that play essential roles in apoptosis and inflammation), induces apoptosis, and prevents eukaryotic elongation factor 2 (eEF-2)-dependent translocation, and (iv) the protein synthesis inhibitors verrucarin A (VrcA) (R_5_) (prevents peptidyl transferase activity in ribosomes). All cytotoxic drugs used were highly active in blocking LNCaP cell proliferation (IC_50_ < 13 nM). For these LTDs, they used either the entire structure of **25** (A_1_) or the linker used in **27** (A_2_) or shorter versions with charged (Glu) and aromatic (Phe) components (A_3_) (Figure 12).

The coupling of the pharmacophore-linker part (DUPA-linker-SH) with the toxophore (A_1–3_-R_1–5_, Figure 12), e.g., in the therapeutic LTD **26** (DUPA-SS-TubH I, DUPA-Aoc-(Phe)_2_-Dap-Asp-Cys-S-S-(CH_2_)_2_OC­(O)-TubH) was accomplished by reacting the DUPA analogue, bearing a free thiol group (A_0_, DUPA-linker-SH), to the activated TubH derivative **20** (Y-R_1_, R_1_ = TubH I (red), Y = (green), leaving group as a pyridine-2­(5*H*)-thione) (Figure 12).^90^ Initially, the methodology was developed for the synthesis of bioconjugate LTDs between TubH and folic acid^130^ and adapted for the PSMA-targeting LTDs **26** and **27**, using the diagnostic **25**.

The efficacy and specificity of the DUPA-based LTDs **26–32** were proven by the inhibition of PSMA­(+) cells with and without the presence of 100-fold excess of PMPA (blocking), while their affinity was measured vs ^99m^Tc-DUPA (**25**) (*K*_d_ = 14 nM),^126,127^ where they showed affinities in the range *K*_d_ = 7–70 nM.^90^ HPLC-MS analysis of LTDs **26–32** post incubation (2–4 h) with GSR (10-fold excess GSR) demonstrated a 98% reduction in disulfide bonds (general mechanism, Figure 8, proposed mechanism for glutathione (GSH), Figure 13). The DUPA-SS-cytotoxic drug conjugates **26–31** showed high cytotoxicity for LNCaP cells with IC_50_ values (IC_50_ = 6–115 nM) in the low nM range except for **32** (*K*_d_ = 34 nM, IC_50_ > 1 μM), which demonstrated only minimal cytotoxicity. The best compounds were DUPA-SS-TubH I (**26**) and DUPA-SS-DAVBH (**29**),^90^ both prodrugs of vinca alkaloids.

**Figure 13:**
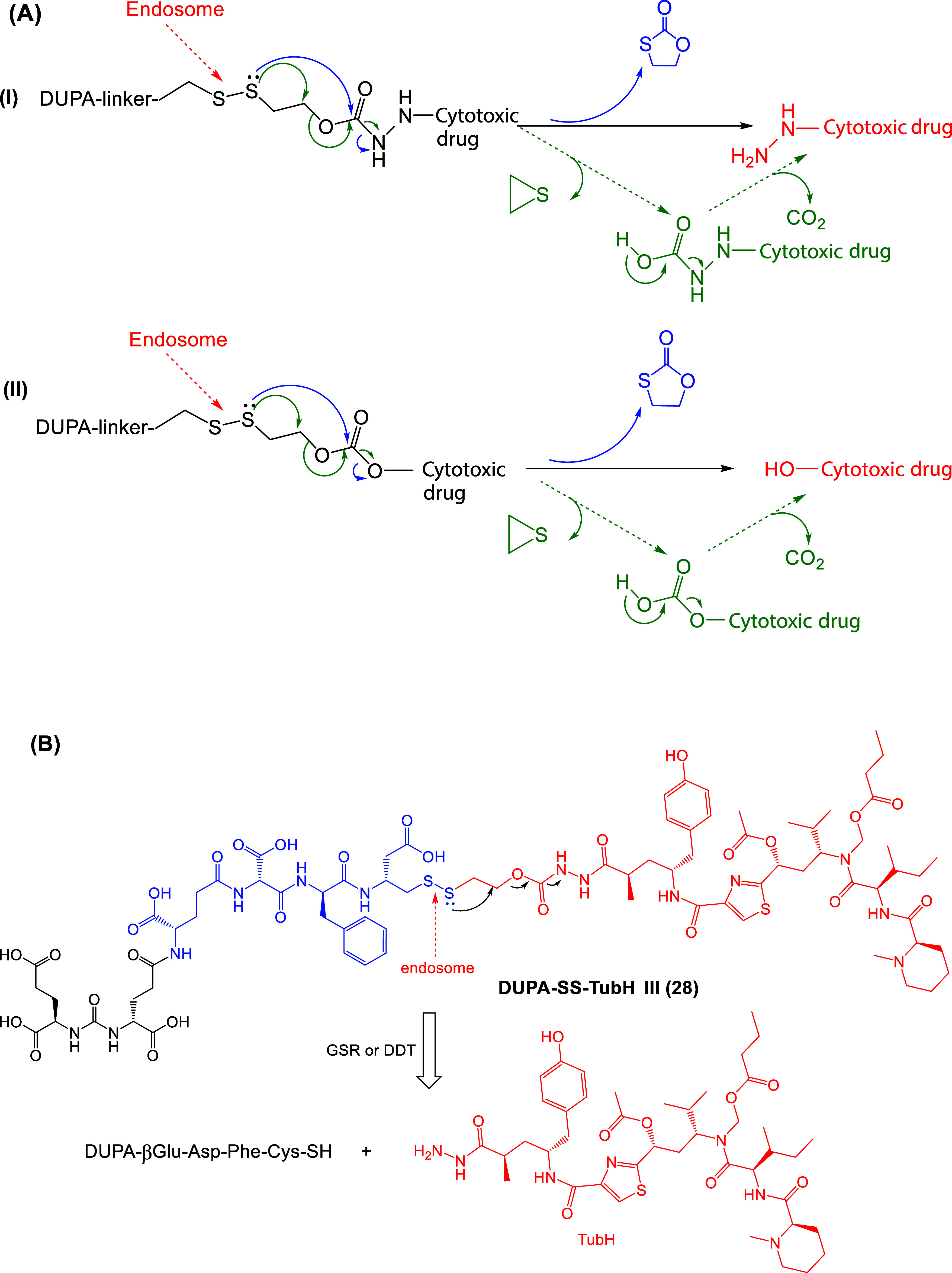
(A) Proposed mechanistic paths for disulfide-mediated release of the cytotoxic drugs from PSMA-targeted self-immolating LTDs inside the cells by the endosome and GSH, which results (I) in the hydrazide or (II) in the alcohol form of the cytotoxic drug, via the ethylene sulfide and carbon dioxide (major route) or 1,3-oxathiolan-2-one (10, minor route) intermediates. (B) An example of release of TubH from DUPA-SS-TubH **28** by GSR or DDT reduction (black: PSMA pharmacophore; blue: linker; red: cytotoxic drug : tubulicine).

Additional results from ref 90 are (a) Cpt, a topoisomerase I inhibitor, which was relatively inactive for LNCaP cells was found to be highly potent as its DUPA-based conjugate **30**. Possibly, because the attached DUPA-linker (at 20-OH) stabilized the lactone ring against hydrolysis, rendering LTD the DUPA-SS-Cpt **30** capable of surviving the 2 h incubation in culture medium without hydrolysis to its inactive form.^90^ (b) The DUPA-SS-VrcA **32** failed to kill LNCaP cells in culture despite the high potency in its nontargeted form (VrcA). This likely happened because of the different routes of cellular entry of VrcA in the targeted and nontargeted forms. After its internalization by PSMA, hydrolases in endosomal compartments can cleave either the epoxide or the lactone ring of VrcA, while the free drug could reach its target location within the cell directly.^90^ (c) LTD of the other protein synthesis inhibitor DidB, DUPA-SS-DidB **31** effectively killed LNCaP cells such as DUPA-SS-Cpt **30**.^90^ (d) For the disulfide-bridged self-immolative DUPA-based LTDs **26–32**, the cytotoxic drug was released after the reduction of the S-S bond inside the cell, while the proposed mechanisms are described in Figure 13.

The stability of the bioconjugates in blood circulation is crucial since it guarantees that the intact form of the bioconjugate would reach the site of activation. DUPA-SS-TubH III (**28**) was stable enough in circulation to reach the PSMA receptor in PSMA­(+) LNCaP tumors in its complete, nonreduced form.^127^ The LC/MS analysis after the exposure of **28** to GSH or dithiothreitol (DTT) (37 °C) showed that >95% of the disulfide bonds produced free TubH via a carbamic acid intermediate (Figure 13). Thus, disulfide bond dissociation within the PCa cell endosome was suggested as the primary route for effective drug release.

As a continuation of previous work, Cushman and collaborators synthesized in 2015 ^131^ the DUPA-SS-indenoisoquinoline (**33**) analogue (Figure 14). In LTD **33**, the DUPA pharmacophore was connected via the linker Aoc-(Phe)_2_-DAP-Asp-Cys designed (same as LTDs **25**) to an indotecan analogue. The design was based on the X-ray structures of PSMA-ligand complexes (i.e., PDB IDs 3BI1,^21^ 2C6C,^19^ 3D7H^26^), which revealed that the used linker could fit in the ∼20 Å deep narrow area connecting the entrance of the funnel with enzyme active site (Figure 10).^16,21,26^ LTD **33** (DUPA-Aoc-(Phe)_2_-Dap-Asp-Cys-S-S-(CH_2_)_2_OC­(O)­O–indotecan) could release an active cytotoxic indotecan molecule inside the cell after cleavage of S-S bond in endosome (self-immolative linker S-S-(CH_2_)_2_OC­(O), Figure 14).^131^

**Figure 14:**
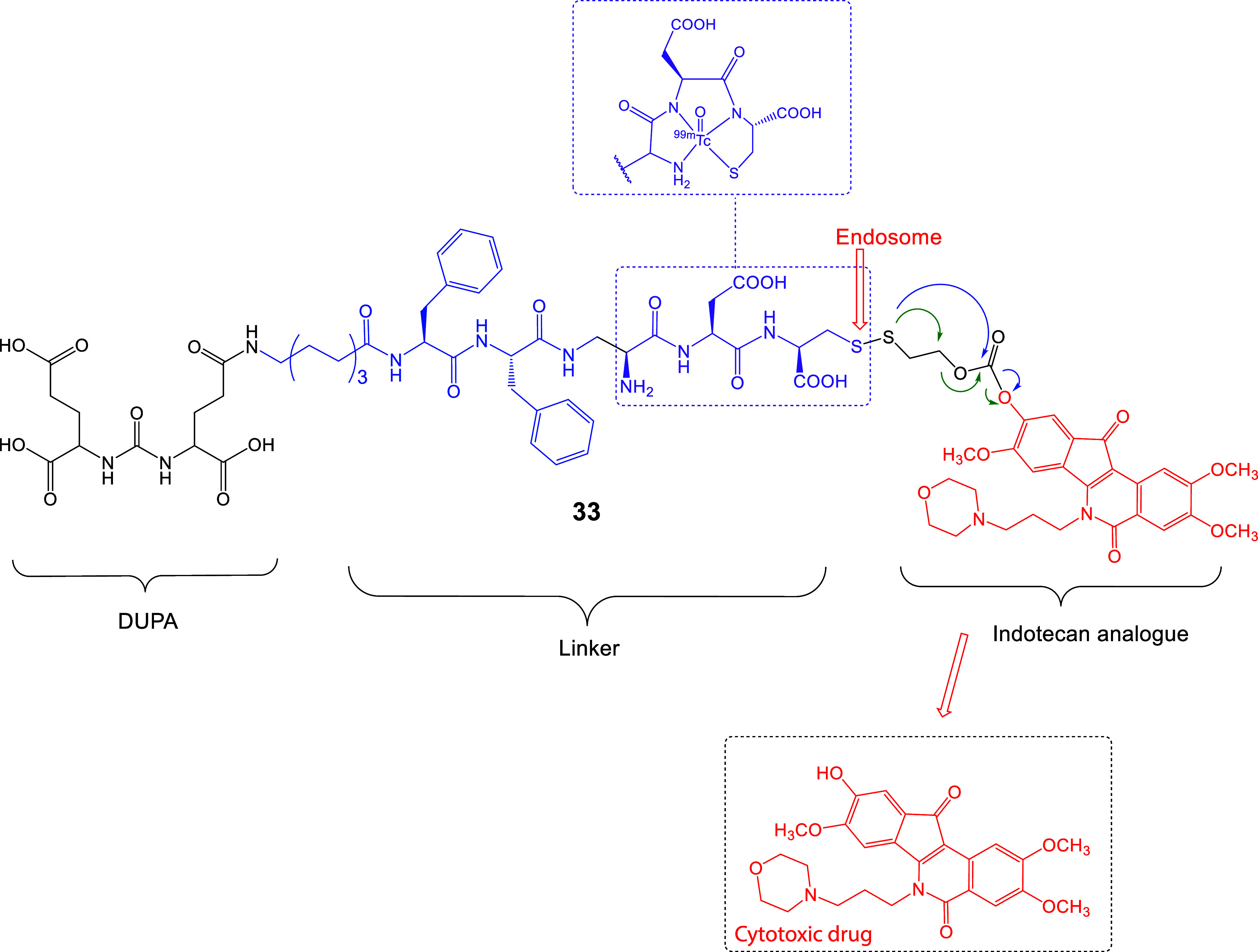
Chemical structure of the LTD (**33**), DUPA-SS-indotecan analogue. The active indenisoquinoline cytotoxic drug (red) can be released inside the cell after dissociation of the S-S bond in endosomes through the disulfide-bridged self-immolative S-S-(CH_2_)_2_OC­(O) linker. Part of the linker was used in analogue **25** for ^99m^Tc complexation (purple) (see also linker A_1_ in Figure 12).

The LTD **33** synthesis could be used as an example synthetic process for linkers, which include the Cys moiety. The use of the Fmoc-H-Cys­(Trt)-(2-ClTrt) resin suppressed racemization of l-Cys to d-Cys, while the (OtBu)_3_-protected DUPA-COOH was coupled to H_2_N-Aoc, at the last step to afford DUPA-Aoc-(Phe)_2_-Dap-Asp-Cys. At the next stage, the DUPA-linker part was coupled with the chemically activated indotecan analogue (2-py-S-S-(CH_2_)_2_OC­(O)­O–indotecan analogue, Figure 12),^131^ as described before in Figure 12.

The biological evaluation of LTD **33** in 22RV1 prostate carcinoma epithelial cells using a competition binding assay with ^3^H-thymidine showed no potency after 2 h of incubation, while the free indenoisoquinoline had an IC_50_ = 2.0 nM. However, LTD **33** after 24 h of incubation produced an IC_50_ = 11.4 nM. This was likely because the mechanism of action for LTD was mediated by the PSMA protein. The efficacy and toxicity of LTD **33** were also studied *in vivo* in 22RV1 xenograft-bearing mice compared with the free indenisoquinoline. A dose of 40 nmol/mouse (2.0 μmol/kg, ip injection) 3 days/week for 3 weeks (9 doses in total) showed complete cessation and regression of tumor growth like the indotecan analogue, while toxicity was selective for PCa cells. In addition, the mice receiving LTD **33** did not show a loss of body weight, and no deaths were noted in contrast to the group treated with the indotecan analogue alone.^131^

The future drug design of indenoisoquinoline–DUPA conjugates is likely to face significant challenges, mainly due to the need for the right solubility characteristics. These are essential to ensure proper formulation and optimize bioavailability at the PSMA action site on PCa cells.^131^ Nevertheless, a few years later, a company named Endocyte, Inc. investigated several LTDs of Tub (hydrazide form, TubH), with EuK (**34–36**, Figure 15) following the previous context.^132^ In the Endocyte’ s LTDs, the pharmacophore DUPA was replaced with EuK and connected to the linker with a more biologically stable urea bond instead of a peptide bond. The linker structure was modified by keeping the essential aromatic parts (*N*-acyl-[4-(aminomethyl)­phenyl]-acetamide) and adding a.a. with COOH pendant arms (Asp)_2_ like LTD **28** (Figure 12) and Cys to form the self-immolative disulfide-based linker, S-S-(CH_2_)_2_OC­(O), which can be cleaved by GSR in endosome. They also tested variations of the EuK pharmacophore vs PMPA, i.e., d-Glu-l-Lys, d-Glu-d-Lys, and l-Glu-d-Lys, which proven to be nonbinders and thus resulting in the EuK (**1**)­as the optimal choice. (Substitution of EuK’s Glu with Asp or Aad (2-aminoadipic acid) reduced PSMA affinity by 17- and 6-fold, respectively, indicating that the extra methylene in the side chain of Glu is optimal for binding the pharmacophoric part to the PSMA binding area.)^131^ The water-soluble conjugate EC1169 (**34**), which had the EuK pharmacophore, presented the highest affinity for PSMA­(+) LNCaP cells, 6.5-fold higher than PMPA. This agreed with structural studies of related ligands and bioisosteric molecules regarding their binding affinity to the binding area of PSMA around the enzymic center.^21,22,26,29,133^ Two other bioconjugates were synthesized, EC1453 (**35**), with a more stable thioether bridge compared with the bioreleasable disulfide bridge, and EC1555 (**36**) lacking the PSMA ligand (control). The synthetic processes used for EC1169 were a combination of liquid chemistry, for the synthesis of EuK, and of solid-phase methods based on the Fmoc-H-Cys­(Trt)-(2-ClTrt) resin for the linker (previously used for **33** ^131^).

**Figure 15:**
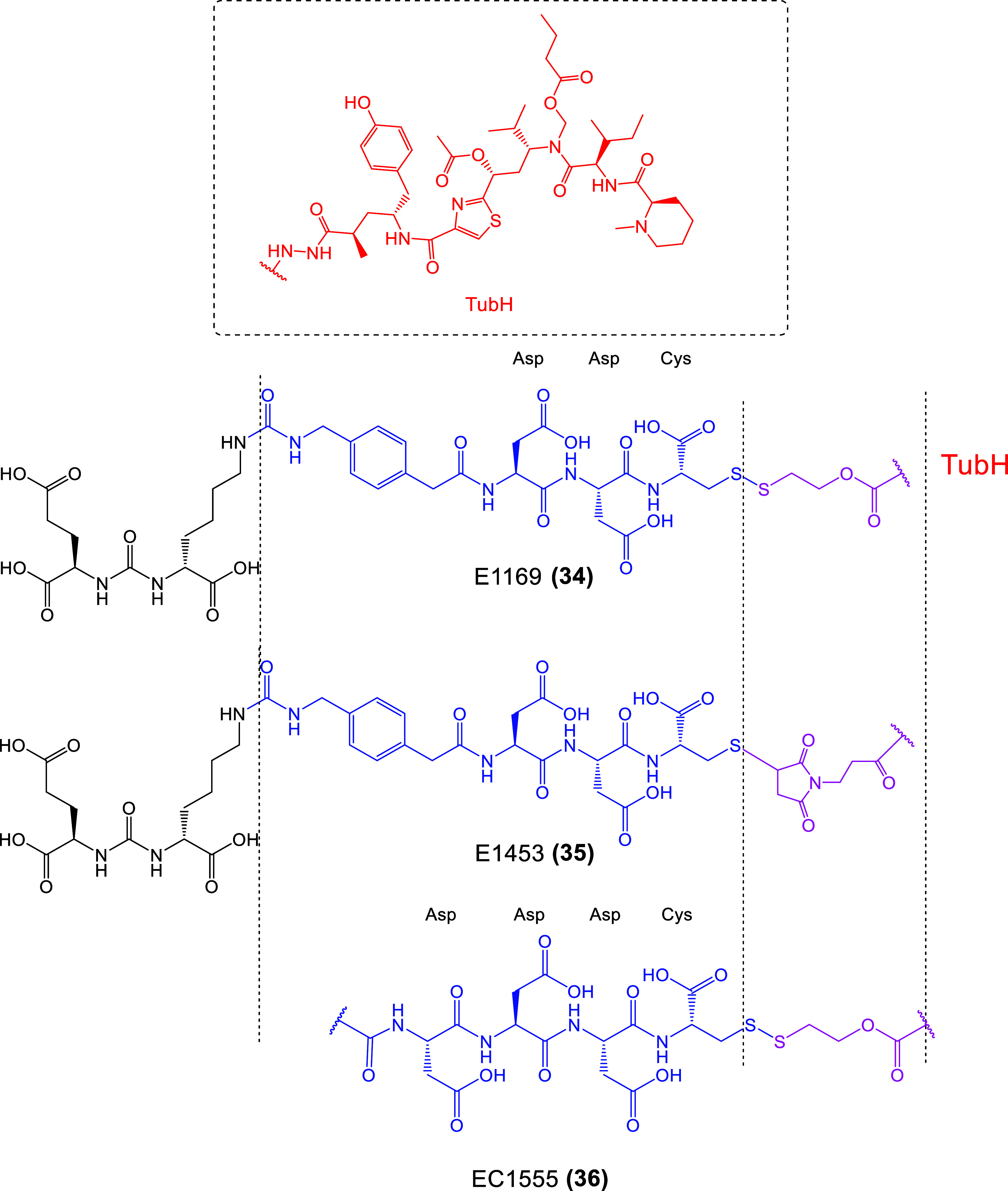
Chemical Structures of LTDs targeting PSMA synthesized as prodrugs of TubH: EuK-SS-TubH (EC1169) (**34**), which releases TubH through GSR in endosome, EC1453 (**35**) with the more stable thioether bridge **4**, and EC1555 (**36**) without a PSMA pharmacophore^132^ (black: PSMA pharmacophore; purple: linker part which can be cleaved or not; blue: linker; red: cytotoxic drug : tubulicine).

The PSMA­(+) LNCaP cells were found to be highly sensitive to EC1169 (**34**); resulting in cell death within 24 h (IC_50_ ∼ 13 nM) after exposure to it, while **34** was inactive against PSMA(−) cells (cells were sensitive to free Tub, IC_50_ = 0.9 nM, A549 human adenocarcinomic, IC_50_ = 0.6 nM, KB human epithelial carcinoma cells). Short-duration treatment of nude mice bearing LNCaP human xenografts with EC1169 (**34**) (*D* = 2 μmol/kg, 3x/W, 2-week schedule) led to complete remissions and cures from cancer (29% tumor-free mice). More importantly, the cancer cytotoxic activity occurred with the absence of weight loss. On the contrary, PSMA(−) KB tumors did not respond to EC1169 (**34**) therapy, confirming its specificity for PSMA. Also, no antitumor activity was observed with EC1555 (**36**) (control without EuK) or when the bioreleasable disulfide linker was replaced with a more stable thioether linkage in EC1453 (**35**). EC1453 (**35**) was inactive *in vivo* against the LNCaP tumor model at doses near to or greater than the maximum tolerated level, while treatment with docetaxel of the LNCaP-bearing mice showed modest antitumor activity, and this was also associated with severe weight loss. Finally, no resistance occurred against EC1169 (**34**) or free Tub drug after the first cycle of therapy. Multiple cycles of **34**-treatment were required for prolonged antitumor responses.^132^

The promising preclinical results of EC1169 (**34**) led to its phase I clinical testing.^134,135^ Preliminary phase 1 (NCT02202447) results of LTD EC1169 (**34**) in men with progressive mCRPC showed promising efficacy. Part A (completed) of the study was about dose-escalation (*D* = 6.5 mg/m^2^; day 1 + 8/28 days), while part B was for the expansion of the study in mCRPC. In these clinical trials, the imaging agent ^99m^Tc-EC0652 ^136^ was also evaluated resulting in excellent tumor localization. Part B of the study included 2 cohorts: C1-mCRPC-taxane not exposed (45 patients) and C2-taxane exposed (40 patients), 34/85 patients were treated (14 patients C1, 20 patients C2) (median age = 70 years, median number of cycles = 3). 76.5% (26/45) of patients reported at least 1 treatment related to adverse effect (TRAE). Most TRAEs were Gr1 and Gr2, and a Gr3 occurred in 1 patient, while Gr4 was not reported. A few dose reductions due to adverse effects had occurred. 6/12 C2 patients had stable disease or better at their first postbaseline scan (9 weeks). One patient currently beyond the 18-week scan had achieved durable resolution of his soft tissue disease. Overall, there was evidence of antitumor activity for both cohorts (C1, C2).^134,135^

Like Tub, paclitaxel (PTX, Figure 16) is a mitotic inhibitor that also targets microtubules and serves as a first-line chemotherapeutic drug for PCa treatment. However, its therapeutic efficacy is significantly limited by its toxicity, resulting from its nonspecific distribution *in vivo*. The LTD **37** was based on DUPA, which was connected to PTX via a cleavable S-S-linker (**37**, Figure 16).^137^ It was hypothesized that the PSMA targeting of **37** would enhance PTX transport ability and tumor selectivity, minimizing adverse effects.^137^ PTX was released *in vitro* after treatment of prodrug **37** with DTT. This was a fast process in contrast to the DUPA-PTX control (connected with an ester bond), which was extremely slow. HRMS studies confirmed the cleavage of **37**’s disulfide bond, which generated a thiol-containing intermediate PTX-SH (Figure 16). Cell viability decreased with increasing concentrations of **37** in the PSMA­(+), 22RV1 and LNCaP cells, and the effect was PSMA specific. No effect was observed for either of the bioconjugates **37** or DUPA-PTX (control with ester bond) in the PSMA(−) PC-3 and HK-2 cells. However, both bioconjugates showed higher IC_50_ values for 22RV1 cells than the original PTX during cell-viability assays (**37**, IC_50_ = 121.1 nM, Control, IC_50_ = 563.3 nM, PTX, IC_50_ = 14.25 nM). It was hypothesized that this effect was due to the slow release of PTX. The control DUPA-PTX (IC_50_ = 563.3 nM) exhibited poor cytotoxicity within the studied range of PTX concentrations, possibly due to the extremely slow hydrolysis rate of PTX, and due to the absence of the self-immolative linker S-S-(CH_2_)_2_OC­(O). This agreed with the PTX concentration in cells incubated with either **37** or free PTX, which were markedly higher.^137^

**Figure 16:**
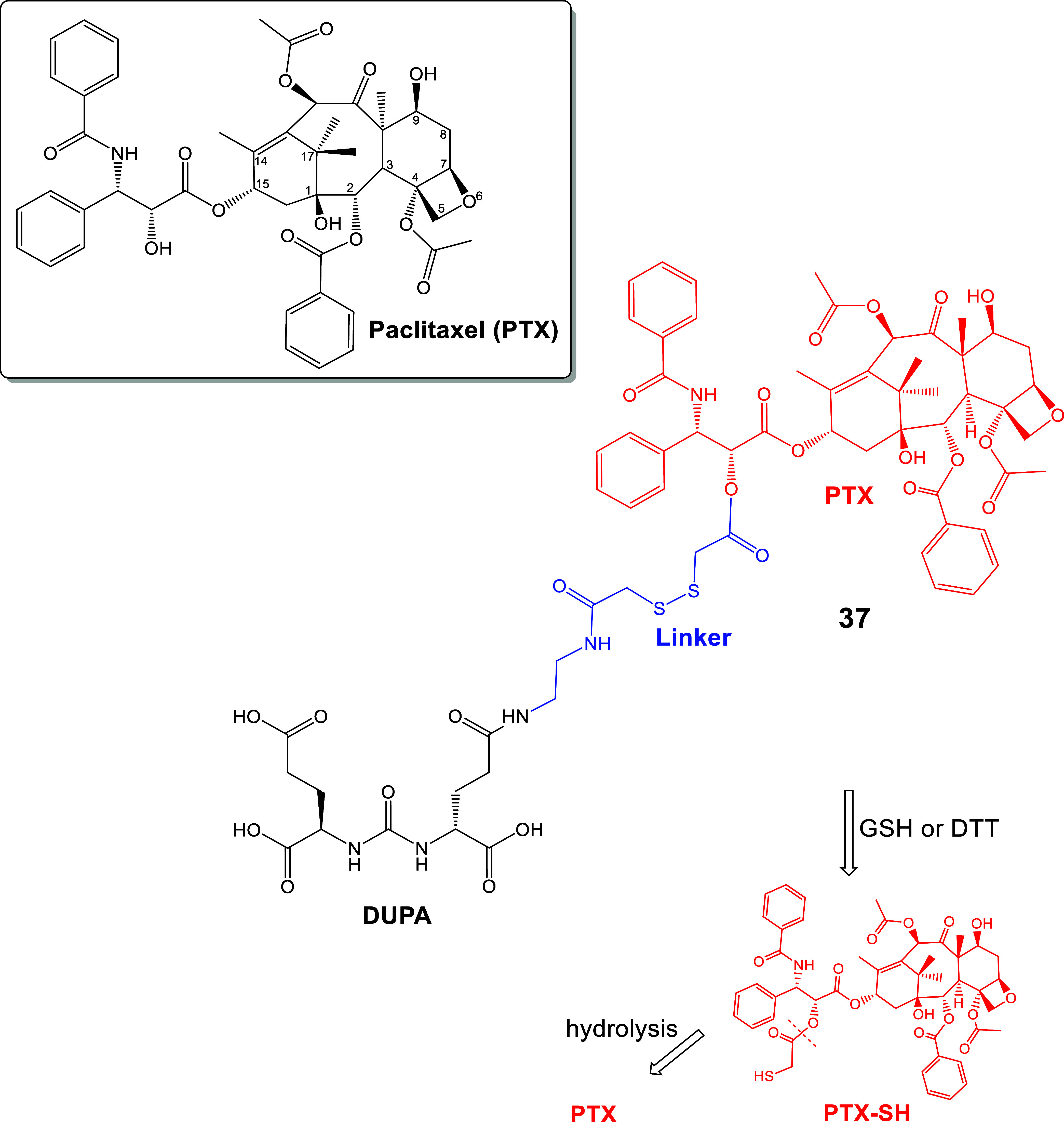
Chemical structure of PTX and LTD (**37**), DUPA-SS-PTX . The activation procedure leads to PTX-SH, where the SH–CH_2_–CO group is easily hydrolyzable in an acidic endosome environment (black: PSMA pharmacophore; blue: linker; red: cytotoxic drug : paclitaxel (PTX)).

*In vivo*, in mice xenograft models bearing 22RV1 tumors, PTX did not show specific tumor targeting, while high PTX contents were noted in the lungs (6.35 μg/g), which eventually led to toxicity in other normal organs. In contrast, for **37**, the concentration of PTX in tumors was higher than that in other organs, indicating the specificity of targeting and effective drug delivery. The repeated administration of LTD **37** (IV every 2 days, 5 injections) resulted in the decrease of tumor volume, at a rate equal to PTX, with less toxicity and without weight loss for the animals.^137^

#### LTDs Containing Protease Cleavable Linkers

Kozikowski and collaborators in 2006 ^138^ synthesized LTD **38** by conjugating a PSMA ligand, through a glutaryl group linker to DOX. In this case, the PSMA pharmacophore was derived by replacing one glutamate moiety of DUPA or the lysine of EuK with *p*-aminophenylalanine (4-amino-l-phenylalanine)^49,69,138^ to fit in the hydrophobic pocket accessory of the S1 site. This pharmacophore was based on previously developed PSMA ligands, i.e., ZJ_24_ and ZJ_17_ ^49^ and the clinically applied diagnostic DCFBC^139^ (**38**, Figure 17). Its chemical synthesis was accomplished by the formation of the urea bond between the Fmoc/allyl-protected 4-amino-l-phenylalanine and allyl-protected Glu. The pharmacophore was then connected to the glutaric acid (GA) linker and to DOX via a peptide bond.^138^ The amino group of the pyranose was selected as the optimal site for conjugation of DOX since it was known that acylation of the amino group present in the pyranose ring of DOX results in a decrease in the cytotoxicity of this drug.^140^ The amide bond between the amino group of DOX and the glutaric acid (GA) linker was hypothesized as a cleavage site by amidases/peptidases such as the prostate surface antigen (PSA), after internalization of SMDC **38**, but not by PSMA, which is extracellular, and would be inhibited by the pharmacophore.^138^ Extracellular PSA served as the activating protease in first-generation LTDs based on Thapsigargin (e.g., compound **17**); however, the most notable advancement was achieved with LTD 19, which selectively targeted PSMA (Figure 10)^116^

**Figure 17:**
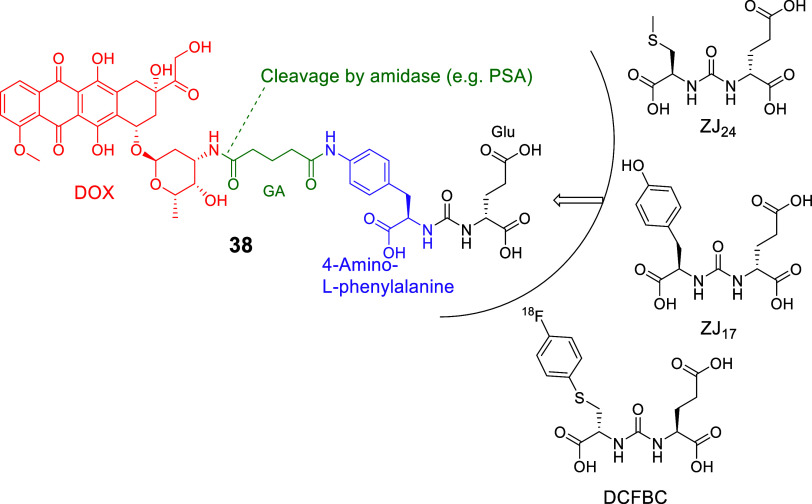
Structure of LTD **38** ^138^ that used a pharmacophore based on the structures of ZJ_17/24_ and DCFBC (black: PSMA pharmacophore; purple: pharmacophore part of ZJ_17/24_ and DCFBC; green: glutaric acid (GA) linker; red: cytotoxic drug ; Doxorubicin (DOX)).

The binding affinity of **38** (Figure 17) was in the nM range (IC_50_ = 40.8 nM vs ^3^H-ZJ_24_, IC_50_ = 15.3 nM). However, its cytotoxicity was less than that of DOX in both PSMA­(+) C4-2 and PSMA(−) PC-3 cells. Despite the selectivity of **38** against C4-2 cells, it was evident that the initial hypothesis for the metabolic cleavage of the peptide bond between glutaric acid and DOX was proven false or it was not effective enough. Thus, the initial hypothesis that SMDC **38** would provide an LTD was not realized.^138^ SMDC **38** is one of the few cases that did not utilize the EuK or DUPA pharmacophore. It would be interesting to see future studies for LTDs with the 4-amino-l-phenylalanine in their pharmacophore in combination with another cleavable linker, e.g., an -S-S- disulfide bridge.

The LTD series **39–43** (Figure 18) is a good example of pharmacophore–toxophore conjugation using copper­(I)-catalyzed azide–alkyne cycloaddition (CuAAC), a widely used bioconjugation reaction from the field of click chemistry. LTD series **39–43** utilizing the EuK pharmacophore and the PTX toxophore were designed and synthesized by Machulkin, Skvortsov, and collaborators in 2019.^141^

**Figure 18:**
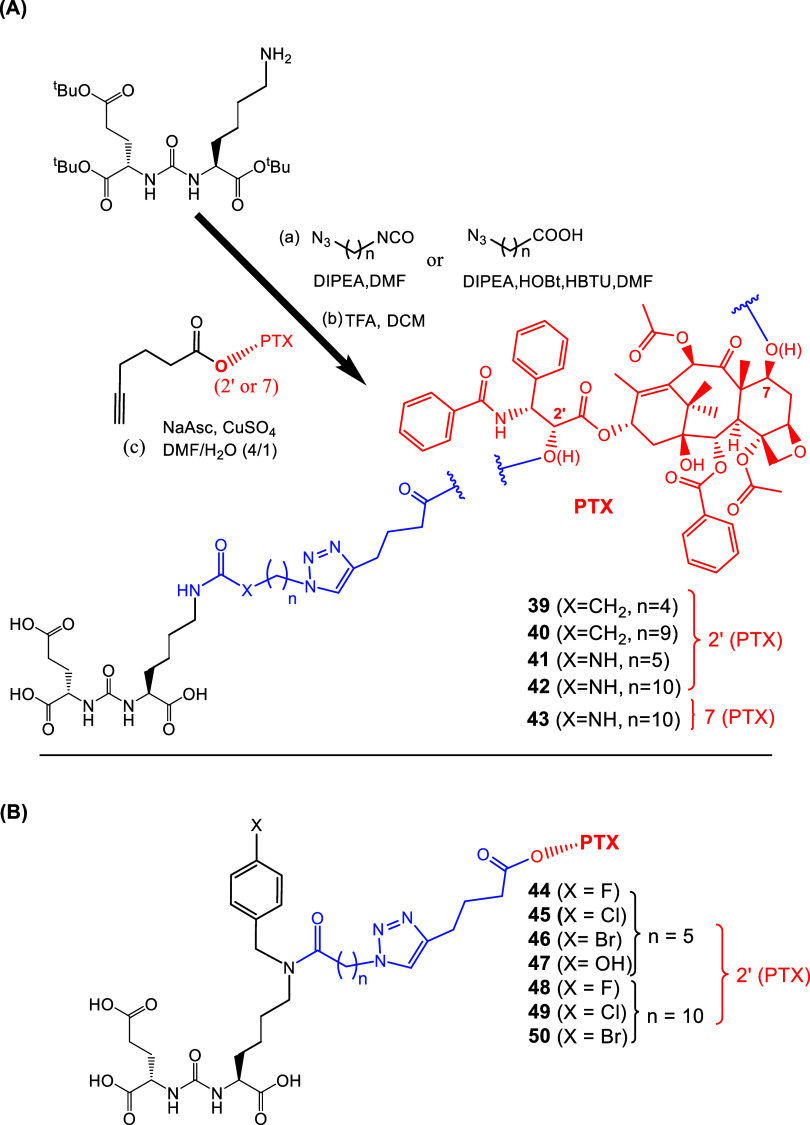
(A) Synthesis of EuK-PTX SMDCs **39–43** targeting PSMA. The EuK pharmacophore was connected via a urea (**39–40**) or an amide (**41–43**) bond to a carbon chain of varied length ending to an azido terminal group, which then reacted to the 5-hexynoic ester derivatives of PTX at 7-OH or 2′-OH via CuAAC reaction. (B) Synthesis of the bioconjugates EuK-PTX **44–50** with aliphatic linkers and benzyl substitution at ε-NH_2_-Lys in EuK pharmacophore, where the 5-hexynoic ester derivative of PTX at 2′-OH was utilized (black: PSMA pharmacophore; blue: linker; red: cytotoxic drug , PTX).

The PTX moiety was connected to either position 2 (**39–42**) or position 7 (**43**). Both cases could release PTX through cleavage of the ester group. However, the 7-OH analogues did not show activity, indicating that 2′-OH position was a better option for conjugation. For the synthesis of **39–43**,^141^ the tBu-protected EuK reacted forming either an amide (**41–43**) or a urea bond (**39–40**) with a carbon chain of varied length with an azido terminal group. The tBu protecting groups of the EuK motif were removed with TFA and then the terminal azido-group reacted with the 5-hexynoic ester derivatives of PTX (at 2′-OH or 7-OH) via CuAAC (Figure 18A) to afford compounds **39–42** or **43**, respectively.^141^

The PTX toxophore could be chemically modified through reaction of its free −OH groups at positions 2′ and 7.^140^ It has been shown that esterification, epimerization, or removal of the 7-OH group in PTX results in insignificant loss of activity, while the 2′-OH group must be free or in a hydrolyzable ester form.^140^ For the 7-OH PTX derivatives, only the EuK derivative with the 11-isocyanoundecanoic acid was used.^141^

Compounds **44–50** (Figure 18B), with aromatic substituents (with a OH- or a halogen at p-position) at ε-NH_2_-Lys in EuK pharmacophore, were developed because of the known influence of para- and meta-substitution with the S1 binding pocket of PSMA,^26,142^ while the C5 or C10 length for the spacer (between tetrazole and urea bond connected to EuK) was selected as the optimal choice based on molecular docking calculations.^141^ The docking calculations were based on the X-ray structure of SMDCs targeting PSMA (PDB ID 4 × 3R).^143^

Compounds **39–43** and **44–50** were evaluated against PCa cells (LNCaP, 22Rv1, and PC-3), noncancerous VA13, and nonprostate (Hek293T, VA13, A549, and MCF-7) cell lines. The most promising compounds of the first series were the 2′-OH LTD **39** (C4 spacer) (MTT assay, LNCaP, IC_50_ = 58 nM, 22Rv1, IC_50_ = 120 nM, and PC-3, IC_50_ = 95 nM, VA13, IC_50_ < 20 nM) and **41** (C5 spacer) (MTT assay, LNCaP, IC_50_ = 13 nM, 22Rv1, IC_50_ = 34 nM, and PC-3, IC_50_ = 25 nM, VA13, IC_50_ = 37 nM), with the latter being more potent, while the 7-OH derivative **43** was not active. The addition of the urea bond in the C5 spacer in **41** was the optimal choice. Compounds without a urea bond and C5 spacer or with a urea bond and a C10 spacer were less active. According to the authors, this was because the urea bond increased the rigidity of the LTD structure. The second series of compounds **47**, **49** (p-OH and p-Cl aromatic substitution, C5 and C10 spacer, respectively) were the most potent (MTT assay, **47**, LNCaP, IC_50_ = 43 nM, 22Rv1, IC_50_ = 61 nM, PC-3, IC_50_ = 90 nM, VA13, IC_50_ = 65 nM; **49**, LNCaP, IC_50_ = 44 nM, 22Rv1, IC_50_ = 120 nM, PC-3, IC_50_ = 20 nM, VA13, IC_50_ = 24 nM). The promising LTD **39** was also examined *in vivo* using 22Rv1 xenograft mice model, where it demonstrated good efficiency comparable to PTX, in combination with reduced toxicity (PTX results for MTT assay, IC_50_ = 3.9 nM, approximately 10× more toxic than **41** *in vitro*). Further studies would be considered for the potent LTDs **41**, **47**, and **49**.^141^

### LTDs with Cathepsin B Cleavable Linkers

#### LTDs Containing the Val-Cit-PAB Linker

Another category of enzymatically cleavable linkers that are often used in PSMA-targeting LTDs include the VCitP motif, cleavable by cathepsin B, a proteolytic enzyme located in the lysosomes.^10,104^ Several examples of prodrugs in this category have utilized the cytotoxic agent MMAE, deriving from peptides called dolastatins, which occur in the marine shell-less mollusk Dolabella auricularia and have the ability to inhibit cell division by blocking the polymerization of tubulin. MMAE has been successfully linked to a monoclonal antibody (brentuximab),^144^ targeting the cell-membrane protein CD30, under the trade name Adcetris. This ADC was licensed against relapsed or refractory Hodgkin lymphoma, systemic anaplastic large cell lymphoma, and T-cell non-Hodgkin lymphoma. Inspired by this story, several groups utilize a PSMA-targeting vector to synthesize LTDs against PCa.

Pomper and collaborators developed in 2021 ^145^ LTD **51** (SBPD-1), with the VCit linker and its analogue **52** (control, SBPD-2) in which EuK was directly conjugated to MMAE ( Figure 19). In **51**, the linker between EuK and MMAE contained an octanodioyl moiety connected with the self-immolative spacer VCit, while in **52** only the octanodioyl-glutaryl motif was included. For the synthesis of (**51**) or the control (**52**), (Glu­(O^t^Bu)_2_-urea-Lys­(O^t^Bu)-DSS) EuK reacted with disuccinimidyl suberate (octanodioate), which was then coupled with the self-immolative VCitP (Val-Cit-PAB) or with a glutaryl motif, respectively. A summary of the synthetic process for **51** is presented in Figure 19

**Figure 19:**
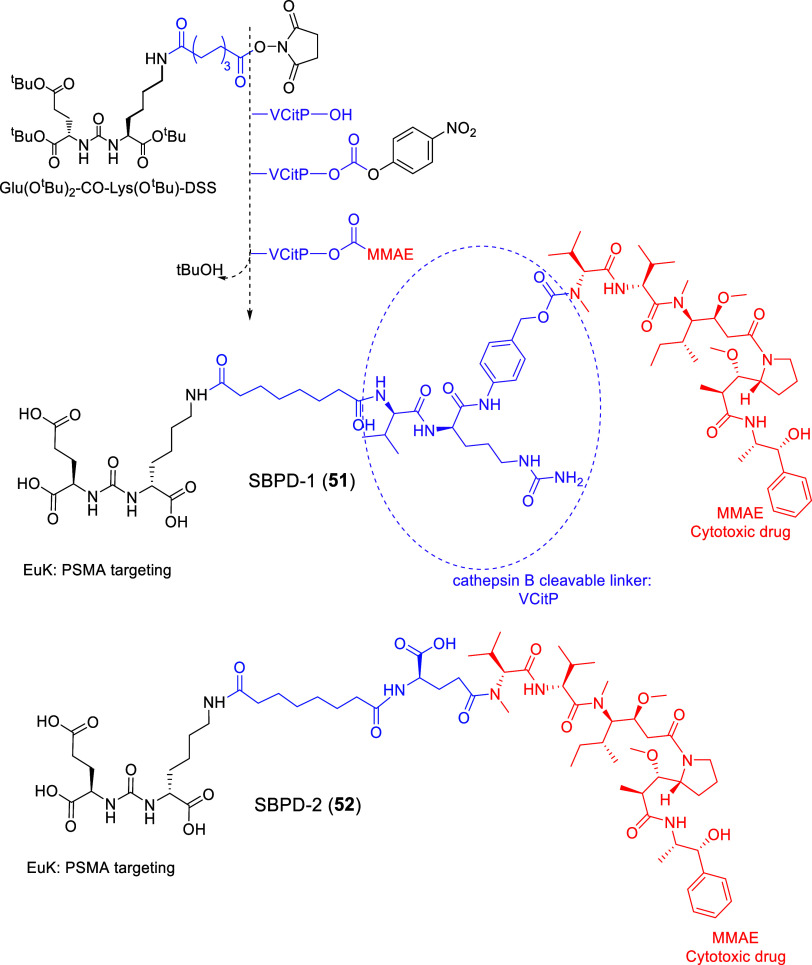
Chemical structures of LTD **51** (SBPD-1), with VCitP a cathepsin B cleavable linker in endosomes and control SMDC **52** (SBPD-2) with a noncleavable linker both conjugated to MMAE (black: PSMA pharmacophore; blue: linker, purple: VCitP; red: cytotoxic drug, MMAE). A summary of the synthetic process for **51** is presented at the top.

Both SMDCs displayed high affinity for PSMA (**51**, *K*_i_ = 8.84 nM, and **52**, *K*_i_ = 3.0 nM). The release of MMAE from LTD **51** was around 80% after 3 h of incubation with recombinant cathepsin B *in vitro*, while it was stable in human plasma. **51** demonstrated selective cytotoxicity against the PSMA­(+) PC-3 PIP cells (IC_50_ = 3.9 nM), while the IC_50_ of the control **52** was in the μM range. MMAE with IC_50_ ∼ 40.0 pM was far more potent than LTD **51**.^145^

The PSMA-targeted prodrug **51** was also tested *in vivo*, where it was found to be effective. The model of testing was immunodeficient mice bearing xenograft PSMA­(+) PC-3 PIP tumors (average tumor volume reached 62.4 ± 11.6 mm^3^), in which several doses of 51 were tested (*D* = 20, 40, and 80 μg/kg, IP/30 days). At the low dose (*D* = 20 μg/kg), **51** was not effective, while at *D* = 40 and 80 μg/kg, it completely cleared the tumors. The median survival period for *D* = 40 and *D* = 80 μg/kg were 15 and 20 days, respectively. **51** was not effective for PSMA(−) tumors, which was indicative of its specificity. LTD **51** also proved effective in an experimental metastatic model of PSMA­(+) PCa (PC-3/ML/PSMA). However, in this case, due to the lower expression of PSMA, higher doses were required (*D* = 80 and 160 μg/kg daily IP/30 days). The prodrug **51** showed significantly less toxicity than MMAE in immunocompetent animals (C57BL/6 mice).^145^

Machulkin and collaborators in 2021 ^146^ utilized a pharmacophore (EuK analogue, IC_50_ = 9 ± 3 nM), established in their previous work with fluorescent dyes^84^ (and very similar to **45** and **49** in Figure 18)^141^ and the linker motif (VCitP) in combination with MMAE to develop **53** (Figure 20).^146^ The structure and conformational features of **53** were studied using 2D NMR. After incubation with cathepsin B, **53** could effectively release MMAE. The biological assays in 22Rv1 PSMA­(+) cells and PC-3 PSMA(−) cells showed a decrease in cytotoxicity for **53** (IC_50_ ∼ 28 and 27 nM in two measurements) in comparison to MMAE (IC_50_ = 1.44 and 0.97 nM).^146^ In addition to microtubule disruption, the authors suggested a mechanism of cytotoxicity for MMAE and its conjugate **53**, which was related to intense oxidative stress. This was supported by *in vitro* experiments, where both MMAE and its conjugate **53** increased reactive oxygen species (ROS) production after incubation with 22Rv1 and PC-3 cells.^146^

**Figure 20:**
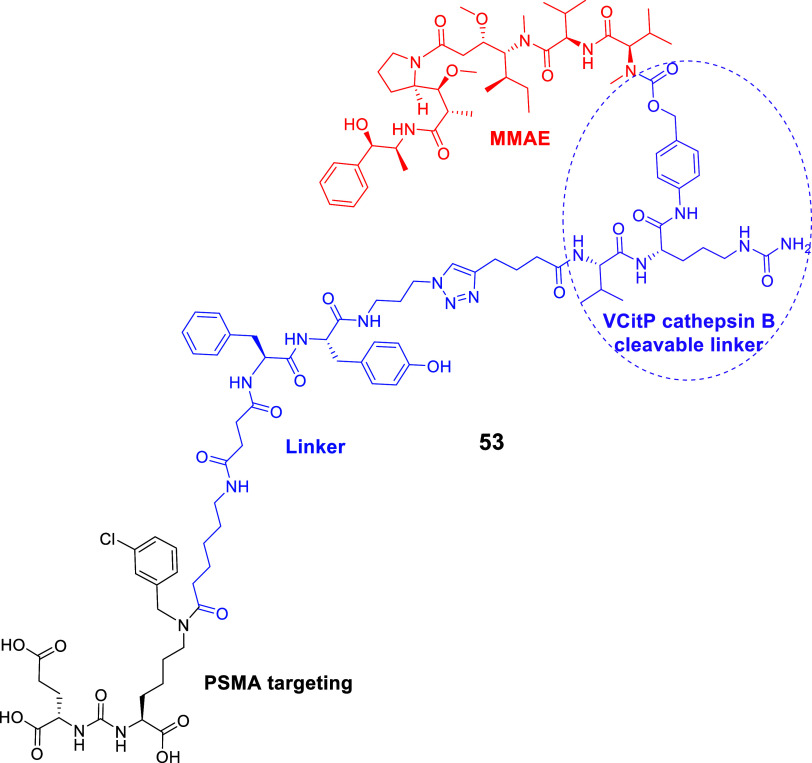
Chemical strucuture of LTD **53**, EuK-VCitP-MMAE For its synthesis they used click chemistry and specifically the CuAAC reaction (black: PSMA pharmacophore; blue: linker; purple: VCitP; red: cytotoxic drug, MMAE).

This was proven to be higher in the case of the PSMA­(+) cell line. Also, based on the mechanism of action of MMAE (microtubule destabilization), a decrease in the average cell stiffness was observed under the action of MMAE or prodrug **53**.^146^

*In vivo* testing of the prodrug **53** in mice and rats (maximum nonlethal dose, MNLD_mice_ = 3 mg/kg, MNLD_rats_ = 4 mg/kg) showed a significant decrease in acute toxicity in comparison with MMAE (MNLD_mice_ = 0.1 mg/kg, MNLD_rats_ = 0.29 mg/kg).^146^ The chronic toxicity of compound **53** was also studied in rats and rabbits. Again, free MMAE was far more toxic than prodrug **53**. Compared with MMAE, **53** proved to be safer for animal survival since all experimental groups of rats survived to the end of the experiment, and among rabbits, only one died and only in the group with the maximum dosage (122 μg/kg). **53** showed visible signs of intoxication only in rats at the maximum dose tested (225 μg/kg). The pathomorphological study revealed pronounced seminal pathology in rats (indicating potential adverse effects on reproductive organs). *In vivo* efficacy evaluation in mice bearing xenograft models showed antitumoral effects in 22Rv1 PSMA­(+) tumors after a single dose of 0.3 mg/kg for the whole duration of the experiment. Triple administration of LTD **53** (3 × 0.3 mg/kg) had no effect on the general condition and weight of the animals, while the average tumor volume increased slowly relative to the average tumor volume of the control group.

The tumor growth inhibition (TGI) for the whole period of observation was 77.4–84.5%. When LTD **53** was applied to the PC-3 PSMA(−) tumor, its antitumor effect was significantly weaker, and TGI for the whole follow-up period did not exceed 37.7%. Also, in general, LTD **53** was less toxic than docetaxel, which was used as a control.^146^

Wang and collaborators in 2021 developed^147^ the bioconjugates **54–56** using DUPA and MMAE (Figure 21). DUPA-Amc-Ahx-(Glu)_3_-Cys-C6-Lys (PSMA-1) in **54** and **55** and DUPA-Amc-Ahx-(Glu)_3_-Cys (PSMA-2) in **56** (Amc = trans-4-aminomethyl-cyclohexanoic acid; C6 = caproyl) were synthesized using solid-phase peptide synthesis (SPPS). The PSMA-1 in **54** was conjugated to MMAE via the Cys residue using the maleimido-C6-VCitP motif or a noncleavable linker (**55**, control for biological testing) and the Lys-C6 motif was either coupled to the dye C6-Cy5.5 (**54**) or not (**56**) (control for biological testing). The dye in SMDCs **54** and **55** allowed their imaging in the biological assays, while the C6-linker was added to optimize the steric hindrance to PSMA during binding to PSMA.

**Figure 21:**
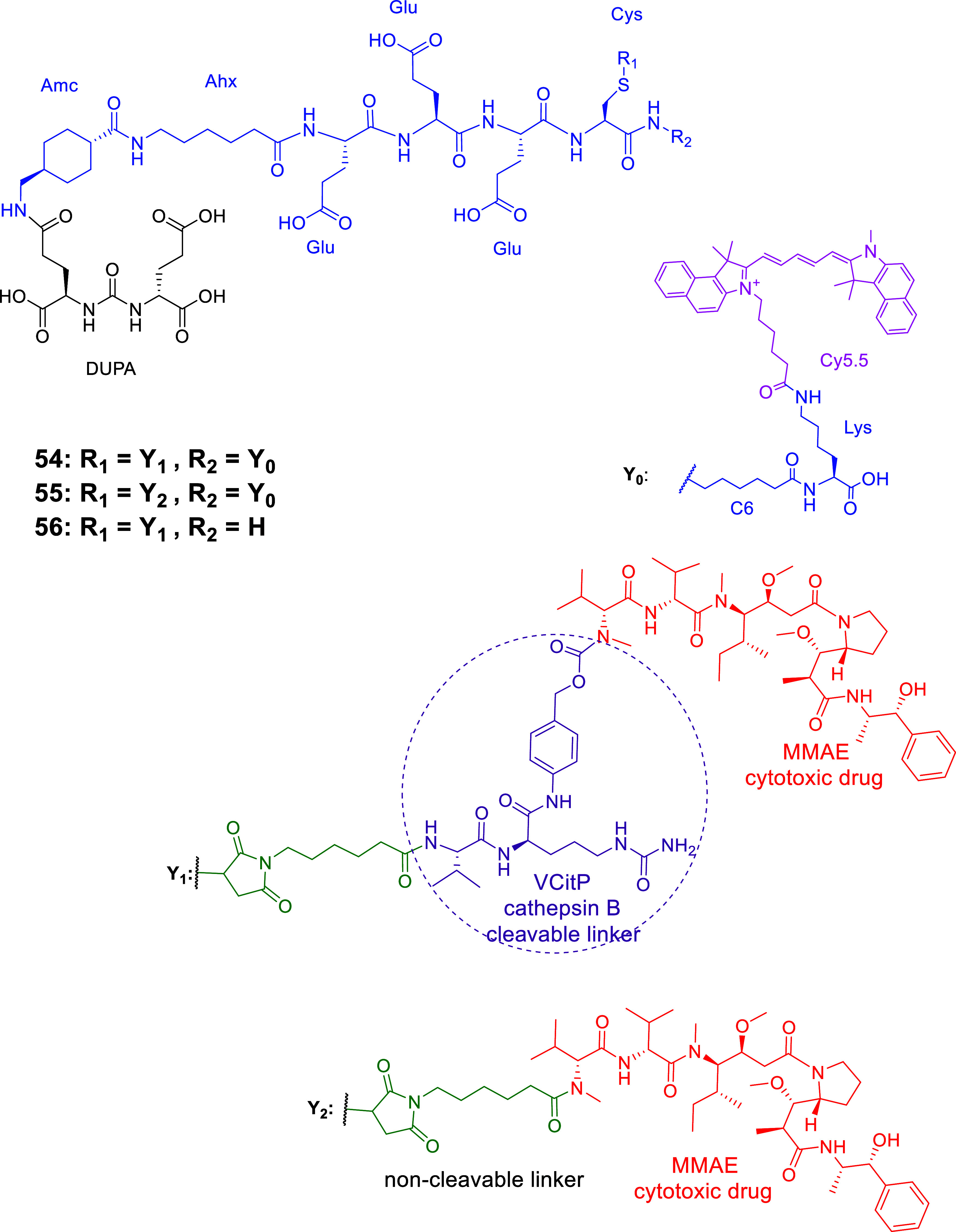
Chemical structures of SMDCs PSMA-1-VCitP-MMAE-Cy5.5 (**54**), PSMA-1-McMMAE-Cy5.5 (**55**), and PSMA-2-VCitP-MMAE (**56**); SMDCs **54** and **56** are LTDs, while **55** has a noncleavable linker with the cytotoxic drug (black: PSMA pharmacophore; green/blue: different parts of the linkers; red: cytotoxic drug, MMAE; purple: dye, Cy5.5).

LTDs showed high affinity values, i.e., **54** (IC_50_ = 3.65 nM, cleavable linker VCitP) and **55** (IC_50_ = 4.88 nM, noncleavable linker). Both LTDs showed affinity values in the range of unconjugated PSMA-1 (IC_50_ = 2.30 nM). It was shown that LTD **54** in the presence of cathepsin B rapidly released MMAE (*t*_1/2_ = 0.33 h) compared with control bioconjugate **55**, which remained intact. LTD **54** and the control SMDC **55** were both internalized showing fluorescence signals localized at the lysosomal compartment of the PSMA­(+) PC-3 PIP cells, while no signal was detected in the PSMA(−) PC-3 Flu cells, or after blocking of the receptor with PSMA-1. Prodrug **54** also affected selective microtubule network disruption in PC-3 PIP causing them to round up and lose the spindle structure, while no such effect was observed for PC-3 Flu PSMA(−) cells or the control. LTD **54** was 20-fold more cytotoxic for the PSMA­(+) PC-3 PIP cells (IC_50_ = 0.84 nM) compared with the PSMA(−) PC-3 flu cells (IC_50_ = 17.0 nM).^147^

*In vivo*, LTD **54** (40 nmol/kg) showed selective uptake in the PSMA­(+) tumors, while at 24 h postinjection there was little to no detectable uptake in PSMA(−) PC-3 Flu tumors. The antitumoral activity of prodrug **54** was also evaluated in mice carrying PSMA­(+) PC-3 PIP tumors (*D* = 160 nmol/kg/animal/4 days—total 5 doses). The average fluorescence signal in PC-3 PIP tumors was similar for LTD **54** and control **55** and peaked at 24 h p.i. (per injection). However, the mice treated with control **55** (having the noncleavable linker) showed the same tumor growth rate as the untreated mice. In addition, prodrug **54** induced more apoptosis in the tumor cells than did the control (**55**) in combination with minimal toxicity. The latter is also indicative of its targeting specificity.^147^

The authors also developed LTD **56** for clinical applications. LTD **56** was structurally similar to **54**, but lacked the Cy5.5 dye component, as it was unnecessary for its clinical application. **56** showed good *in vitro* results, i.e., similar binding with LTD **54** (IC_50_ = 4.34 nM) and 48-fold more potency for killing PC-3 PIP cells (IC_50_ = 4.64 nM) than PC-3 Flu cells.^147^ When LTD **56** was compared with a PSMA-targeting antibody-MMAE conjugate (which has entered clinical trials but failed due to toxicity), it showed less potency regarding PC-3 PIP cells. However, **56** showed a 10-fold or greater safety, i.e., higher maximum tolerated dose (MTD), compared with all of the other MMAE drug forms, i.e., the free drug MMAE and the PSMA ligand-ADC based on MMAE, after a single dose IV in tumor-free athymic nude mice, and higher therapeutic index (20 vs 1, 12.8, respectively). Treatment with LTD **56** exhibited the ability to inhibit tumor growth and prolong animal survival in a dose-dependent manner. At the lowest dose (191 nmol/kg), significant tumor inhibition was observed, while at the highest dose tested (3820 nmol/kg, half the value of its MTD), all five mice survived the 90-day experimental time and three out of five mice were tumor free, resulting in 60% cure.^147^ This direct comparison of the two bioconjugate categories favors peptide targeting over Abs, which showed an improved therapeutic index. The cause of that originates in its PK profile. The smaller **56** had a shorter circulation time and a rapid body excretion, which resulted in less off-target drug activation and reduced toxicity.^147^ In addition to those reasons, one should also mention that peptides show better tumor infiltration and therefore cell internalization, which is a crucial factor for the release of MMAE inside the cell by cathepsin B.

Based on the promising results of the bifunctional molecules (BFMs), Pal et al. developed^111^ a new platform of trifunctional (TFMs) LTDs targeting PSMA (**57–59**, Figure 22). TFMs utilized the EuK connected via a VCitP linker to MMAE or Cy7 and via triazole to an AG10 (Acoramidis) analog. AG10 is a novel, potent, and selective oral transthyretin stabilizer, which was developed to treat transthyretin amyloid cardiomyopathy (ATTR-CM) (currently in Phase III clinical trials).^148,149^ It was hypothesized that the hydrophilic spacers in the transthyretin protein (TTR, a transport protein found in plasma and cerebrospinal fluid) ligands will maintain the overall hydrophilicity of TFMs, limiting their passive diffusion into PSMA(−) cells.

**Figure 22:**
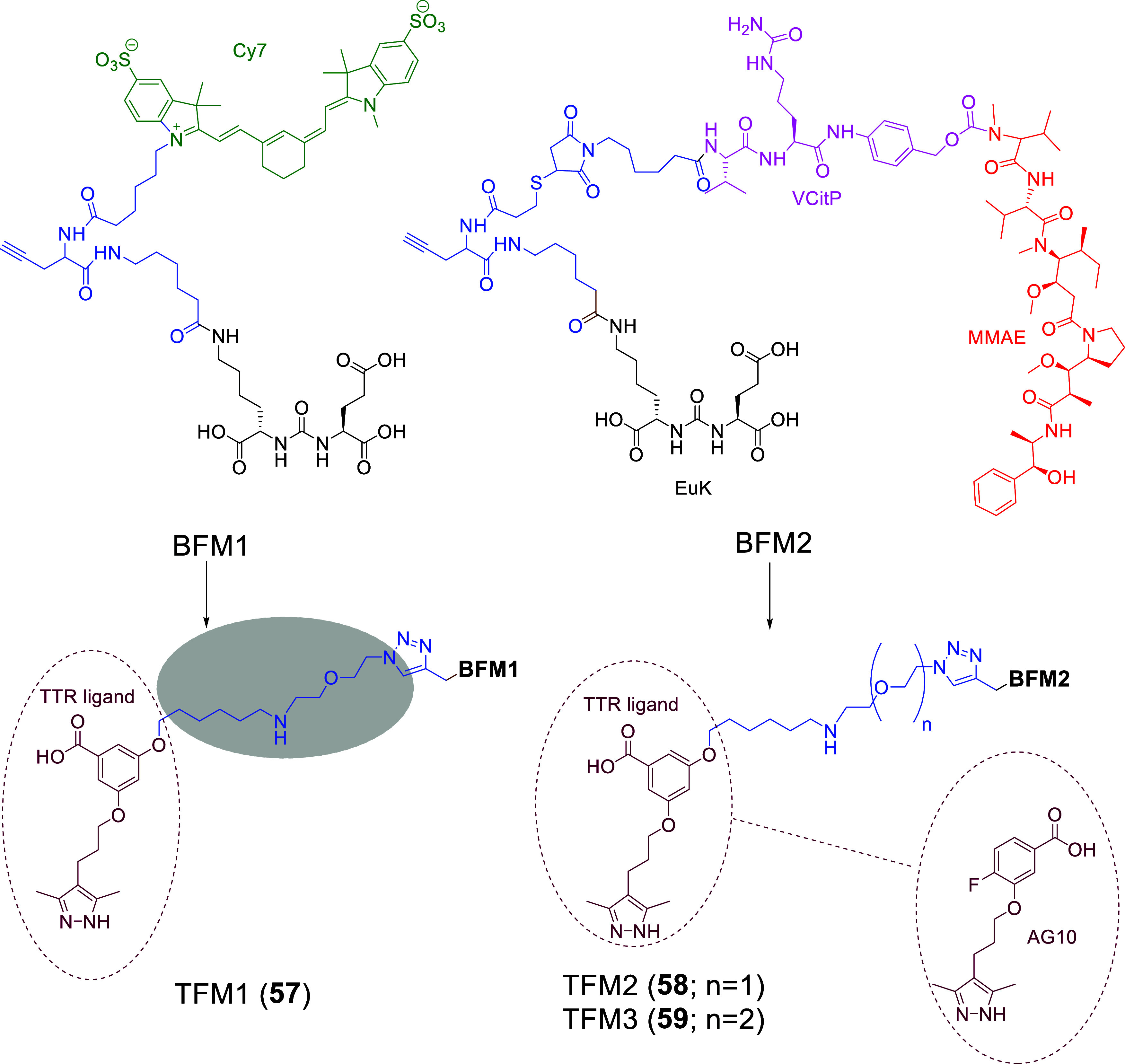
Chemical structures of TFMs **57–59** and BFM precursors, AG10, and its analogue used in SMDCs **57–59** as TTR ligand. TFMs are composed of four modules: the TTR ligand (purple), the EuK pharmacophore (black), the payload (Cy7 green or MMAE red), and the linker system (blue) and VCitP (magenta). The essential amine group in the TTR ligand is highlighted in gray. BFM1 and TFM1 (**57**) incorporate the imaging dye Cy7; attached through a noncleavable linker, therefore, only SMDCs **58** and **59** were LTDs. MMAE was incorporated in BFM2, TFM2 (**58**), and TFM3 (**59**) via the VCitP linker, which is cleavable by cathepsin B. TFM3 (**59**) has a slightly longer PEG spacer that increases its hydrophilicity compared with TFM2 (**58**).

To assess the linker positions of the TTR ligands, which were amenable for modification, molecular docking calculations were performed to identify the interactions between the modified hydrophilic AG10 analogs and the T4 pocket of TTR. Based on the molecular docking calculations, the three TFMs **57–59** were synthesized (Figure 22). The length between the proximal end of the EuK and TTR ligands was optimized to achieve an unobstructed binding to both proteins PSMA and TTR.^111^ TFM1 (**57**) was developed for imaging/diagnostic purposes and had the dye Cy7 attached through a noncleavable linker.^111^ As it was argued, the addition of an amino group in the linker, being protonated at physiological pH, may form salt bridges with the two glutamic acid residues (Glu54 and Glu54′) close to the surface of TTR and can increase the hydrophilicity of the new TTR ligands. The introduction of the amino group (Figure 22, highlighted area) on the linker structure not only increased the affinity and selectivity to TTR but also decreased nonspecific interactions with other serum proteins such as albumin. The synthesis of SMDCs was realized by the reaction of EuK-C_2_H (O^t^Bu protected) with the other two parts: Initially, the (Cy7-NHS) as an *N*-hydroxysuccinimide ester, and then (TTR-N_3_) as an azide (CuAAC reaction), while all of the remaining protecting groups were removed at the final stage with TFA.^111^

TFM1 (**57**), TFM2 (**58**), and TFM3 (**59**) showed lower binding affinities and binding selectivity and occupancy to TTR in serum than the original TTR ligand (TFM1, *K*_i_ = 374.1 nM, TFM2, *K*_i_ = 497.7 nM, TFM3, *K*_i_ = 553.4 nM), possibly because of their larger size in comparison to the original TTR ligands and binding with other proteins. However, TFMs **57–59** showed much higher binding affinity for PSMA (TFM1, *K*_i_ = 33.8 nM, TFM2, *K*_i_ = 7.2 nM, TFM3, *K*_i_ = 14.7 nM), which would enable them to leave TTR in serum and bind to PSMA on the surface of PCa cells. A simultaneous binding to both PSMA and TTR was not anticipated because the PSMA affinity values of **57–59** did not change much after the addition of TTR in the competition studies. The pharmacokinetic studies in rats (single IV dose = 0.1 μmol/kg) verified the initial hypothesis that the polar TTR ligand and linker would improve the pharmacokinetic properties. The results showed significantly higher concentrations in plasma for TFM1 (**57**) (extended circulation) than for BFM1 at all times tested. While there was no measurable amount of BFM1 after 4 h, TFM1 (**57**) was still present even after 24 h. Whole body imaging studies in mice after injection verified the above result showing that the fluorescence signal of BFM1 was significantly reduced at 4 h postinjection reaching background level at 24 h, while TFM1 (**57**) showed a high fluorescence signal at 4 h, which was maintained for up to 48 h postinjection.^111^

It was observed that TFM2 (**58**) and TFM3 (**59**) efficiently released MMAE in buffer, while their selective cytotoxicity was also proved *in vitro*. Specifically, the cytotoxicity of **58** and **59** on PSMA­(+) LNCaP cells (IC_50_ = 5.4 and 3.5 nM, respectively) was much higher than for PCMA(−) DU145 cells (IC_50_ = 313 and 243 nM, respectively). Among the investigated TFMs **57–59**, the more hydrophilic **59** showed the lowest activity against PCMA(−) DU145 cells than PSMA­(+) LNCaP cells (possibly due to the lower passive diffusion across the cell membrane) and was selected for further evaluation. LTD **59** was stable in buffer, human serum, and stable enough in rats and mice serum, while circulation time in rats was increased in the presence of TTR.^111^

*In vivo*, LTD **59** (*D* = 300 nmol/kg/3 days; total 4 doses) effectively suppressed tumor growth of the LNCaP tumors in mice after the third dose (∼70% decrease in tumor volume). The antitumor activity of LTD **59** was significantly (*p* ≤ 0.001) higher than those of BFM2 and free MMAE, while tumor size did not change significantly up to the end of the studied period. The antitumoral activity of free MMAE was lower than both BFM2 and TFM2 (**58**), which was attributed to MMAE’s higher lipophilicity resulting in a higher volume of distribution and lower effective dose reaching the tumor compared with **59**. In addition, MMAE and BFM2 (dose = 600 nmol/kg) were more toxic and had more side effects (dramatic decrease in animal body weight) than **59**.^111^

### Prodrugs with Glu-Val-Cit-PAB Linker

A disadvantage of VCit dipeptide-bearing bioconjugates was that, while VCit was stable in human plasma, it was unstable in mouse plasma, due to extracellular hydrolysis of VCit in mice by the carboxylesterase 1C enzyme.^150^ This makes translating efficacy data from mouse to human challenging. In this regard, Alhamadsheh and collaborators in 2022,^110^ as a continuation to their previous work with TFMs in ref 111, replaced the dipeptide VCit part in the linker with the tripeptide Glu-Val-Cit (EVCit), which was previously reported by Tsuchikama and collaborators in 2018 ^151^ to enhance the stability of ADCs in mouse serum. The new LTD EVCitP-TFM (**60**) (Figure 23)^110^ was an analogue of **59** (Figure 22) with the addition of Glu in the linker (EVCitP).^111^

**Figure 23:**
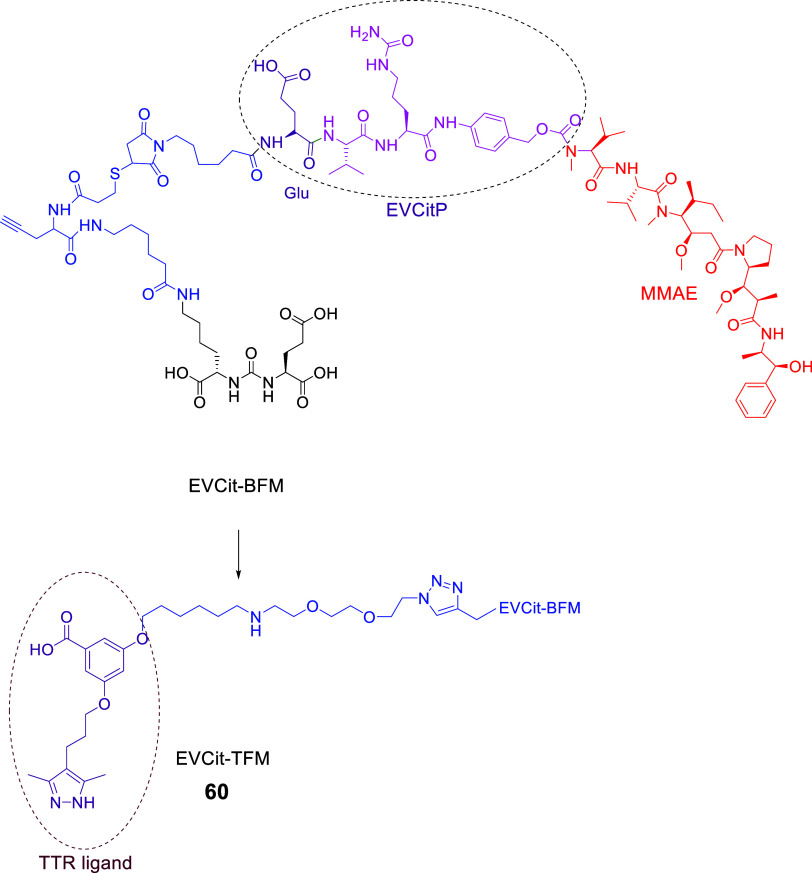
Chemical structures of EVCitP-BFM and EVCitP-TFM (**60**) (TTR ligand (magenta), PSMA ligand (black), cytotoxic drug, MMAE (red), and linker system (EVCitP: purple, Glu: dark purple)).

The authors performed biological experiments to verify the efficacy of **60** in comparison with several VCitP-TFM controls and EVCitP-BFM (control without the TTR ligand).^110^ The EVCitP linker was efficiently cleaved (30 min) by cathepsin B, releasing free MMAE. LTD **60** also showed selective cytotoxicity against PSMA­(+) LNCaP cells, in contrast to PCMA(−) DU145 cells. MMAE showed nonselective cytotoxicity toward both PSMA­(+) LNCaP and PSMA(−) DU145 cells, in contrast to **60** and **59** which were selective for PSMA­(+) LNCaP cells. The selectivity for TTR was evaluated in the PSMA(−) HeLa cell line, where MMAE displayed nonselective cytotoxicity. Both EVCitP-TFM (**60**) and its VCitP-TFM analogue (**59**) displayed ∼3-fold lower cytotoxicity toward HeLa cells in the presence of TTR. There was no effect of TTR on the cytotoxicity profile of EVCitP-BFM (control without the TTR ligand).^110^

The *in vivo* testing in CD-1 mice (single dose intraperitoneal *D* = 1200 nmol/kg/5 days, total = 4 doses) revealed significantly less toxicity for EVCitP-TFM (**60**) and EVCit-BFM in comparison to MMAE and the VCitP-TFM analogue **59** (**59**: *D* = 3.0 mg/kg, **60**: *D* = 3.1 mg/kg, EVCitP-BFM: *D* = 2.5 mg/kg, and MMAE: *D* = 0.4 mg/kg—equivalent to 600 nmol/kg). Dose-escalation study for EVCitP-TFM (**60**), EVCitP-BFM, and docetaxel in male athymic nude mice showed that multiple doses of (**60**) and EVCitP-BFM (*D* = 1200 nmol/kg) can be given to mice without affecting their body weight.^110^ The antitumoral efficacy of EVCitP-TFM (**60**), EVCit-BFM (control), and docetaxel (standard therapy) was tested in PSMA­(+) LNCaP xenograft mouse models. All compounds effectively suppressed tumor growth and delayed tumor relapse; however, mice given LTD **60** continued to gain weight throughout the study. This supported the hypothesis that improving the stability of the linker using EVCitP instead of VCitP improved LTD’s **60** safety profile while increasing its antitumor efficacy *in vivo*. In contrast, docetaxel (*D* = 10 mg/kg), while not being toxic, was not as effective as the two LTDs EVCitP-TFM (**60**) and EVCitP-BFM.^110^

### LTDs with Chemically Cleavable Linkers

#### Prodrugs with Hydrazone-Based Linkers

The hydrazone bond (C = N–NH) is a chemically cleavable linker by hydrolysis at acidic pH that has been used in the development of LTDs. The synthesis of hydrazones is usually accomplished via the nucleophilic attack of amine groups on the electrophilic carbon atoms of an aldehyde or ketone to form a Schiff base. Hydrazones exhibit better intrinsic stability compared with imines, while acylhydrazones show higher hydrolytic stability compared with hydrazones and oximes.^152^ However, hydrazones can be cleaved in the acidic environment of endosomes (pH = 5.5–6.2) and lysosomes (pH = 4.5–5.0) as described in Figure 8.^153^

The synthesis of the DOX-based LTDs **61** and **62** (Figure 24) targeting PSMA was accomplished by connecting the EuK via linkers of various lengths using standard peptide chemistry.^153^ For linker construction, they used 6-aminohexanoic acid (R_1_, *n* = 5) (**61**) and 11-aminoundecanoic acid (R_2_, *n* = 10) (**62**), either connected with adipic acid (example of linker synthesis, Figure 24) or with an aromatic motif −(Phe)_2_– (**63**). LTDs **61–63** were conjugated to DOX via a hydrazone link (final step of synthesis), while for the control (**64**) this was replaced by a peptide bond. SMDC **64** with a noncleavable linker was synthesized in a one-pot reaction. At this point, one should notice the structure similarities with the previously mentioned (**38**),^138^ which failed to release DOX (**61–64**, Figure 24).

**Figure 24:**
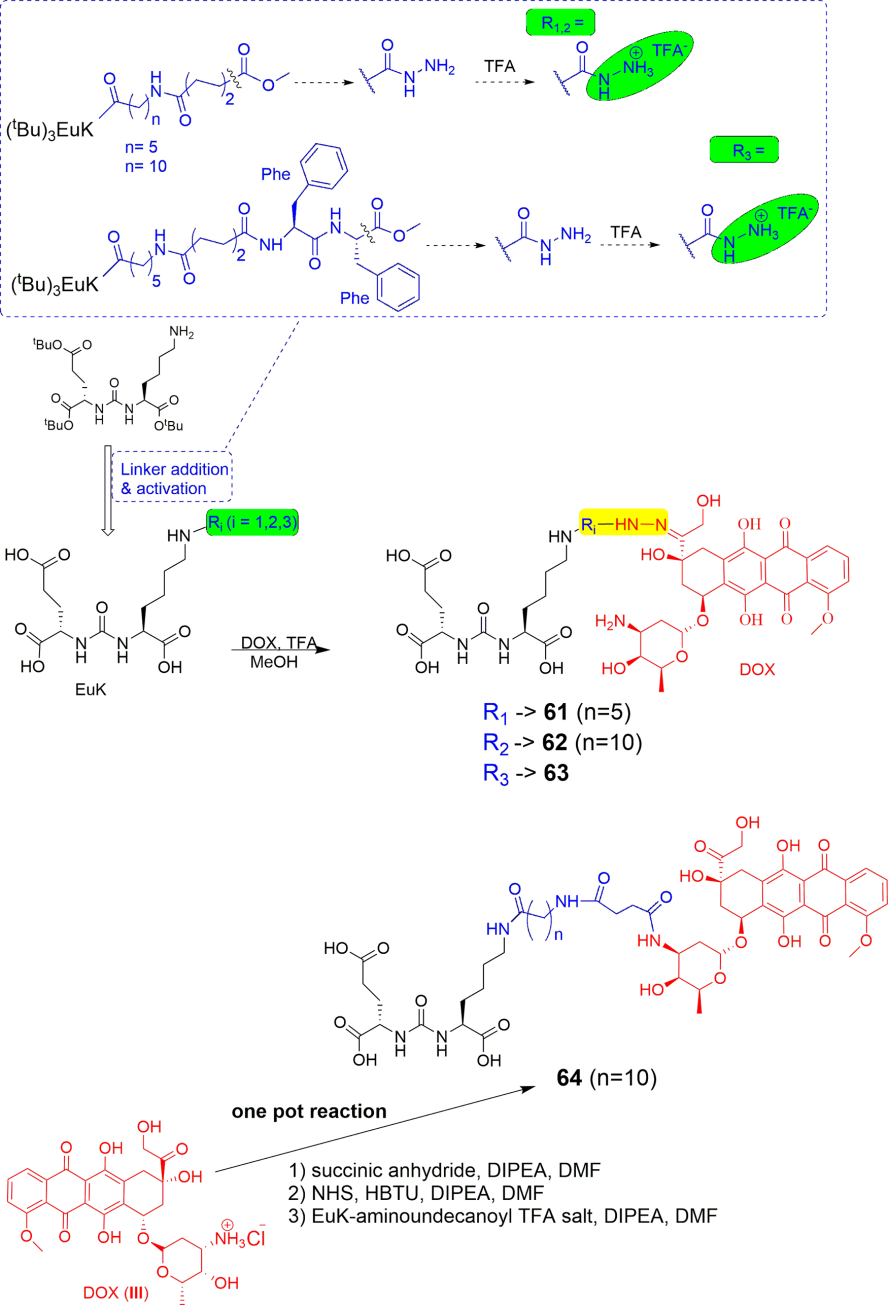
Chemical structure of PSMA-targeting SMDCs **61–64** in which EuK was coupled with DOX (shown in red) using various linkers (shown in blue). The various linkers (R_1,2,3_) used are presented at the top of the figure. In SMDCs **61**-**63**, an acyl-hydrazone bond was formed between DOX and the linker’s terminal hydrazine group (yellow highlight). SMDCs **61**-**63** are LTDs since they can release DOX after cleavage of the *N*-acetyl-hydrazone bond. In control SMDC **64**, an amide was formed between the DOX amino group and linker’s adipoyl moiety.

*In vitro* evaluation based on the fluorescence signal caused by the DOX release showed that LTD **61**, with the shorter linker, was predominately deposited in nuclei and within the cytoplasm, while LTD **62**, with the longer linker, was spread smoothly mainly beyond nuclei. However, LTD **63** equipped by −(Phe)_2_– showed the optimal compartmentalization (primary in the nuclei of LNCaP cells) and selectivity compared with DOX. SMDC **64** with the covalent linker did not release DOX at all. The anticancer potency evaluation of all of the SMDCs **61–64** in LNCaP and PC-3 cells showed that the most active compound was the −(Phe)_2_– analogue **63** (IC_50_ = 95 nM) close to that observed for DOX (IC_50_ = 93 nM). All compounds showed much higher IC_50_ values for PSMA(−) PC-3 cells. Docking calculations based on the X-ray structure of PSMA/GCPII in complex with the ^18^F-labeled phosphoramidate peptidomimetic inhibitor CTT1057 (PDB ID 4JYW^154^) revealed a favorable binding of the long chain linker LTD **62** with PSMA binding area.

*In vivo* biological testing was performed for the most potent LTD **63** and DOX in nu/nu mice with PSMA­(+) PC-3 PIP xenograft after ip administration (*D* = 4 mg/kg/week for 3 weeks). The mice treated with **63** have demonstrated improved food consumption and less weight loss; however, DOX showed better results regarding tumor growth inhibition (TGI^av^ ∼ 92%), in comparison with LTD **63** (TGI^av^ ∼ 65%).^153^

#### SMDCs with Noncleavable Linkers

Denmeade and collaborators in 2021 ^43^ combined EuK pharmacophore with two cytotoxic proteins (**65** and **66**, Figure 25): (a) the human Granzyme B (GZMB-MU2), a serine protease secreted by activated cytotoxic T lymphocytes, which can cleave a myriad of cytoplasmic proapoptotic and antisurvival substrates inducing cell death and (b) a cysteine-containing fragment of the pseudomonas exotoxin A gene (PE35-MU2), which was an engineered version of pseudomonas exotoxin A. This protein fragment despite its inability to bind to cells can disrupt target cell translation via ADP-ribosylation of the crucial diphthamide residue on eEF-2 inhibiting general protein translation. For the preparation of the bioconjugates **65** and **66**, EuK reacted with maleimido-PEG_2_-NHS and the product reacted with the free SH-bearing protein forming an EuK-succinimidyl-S-protein bond (Figure 25).

**Figure 25:**
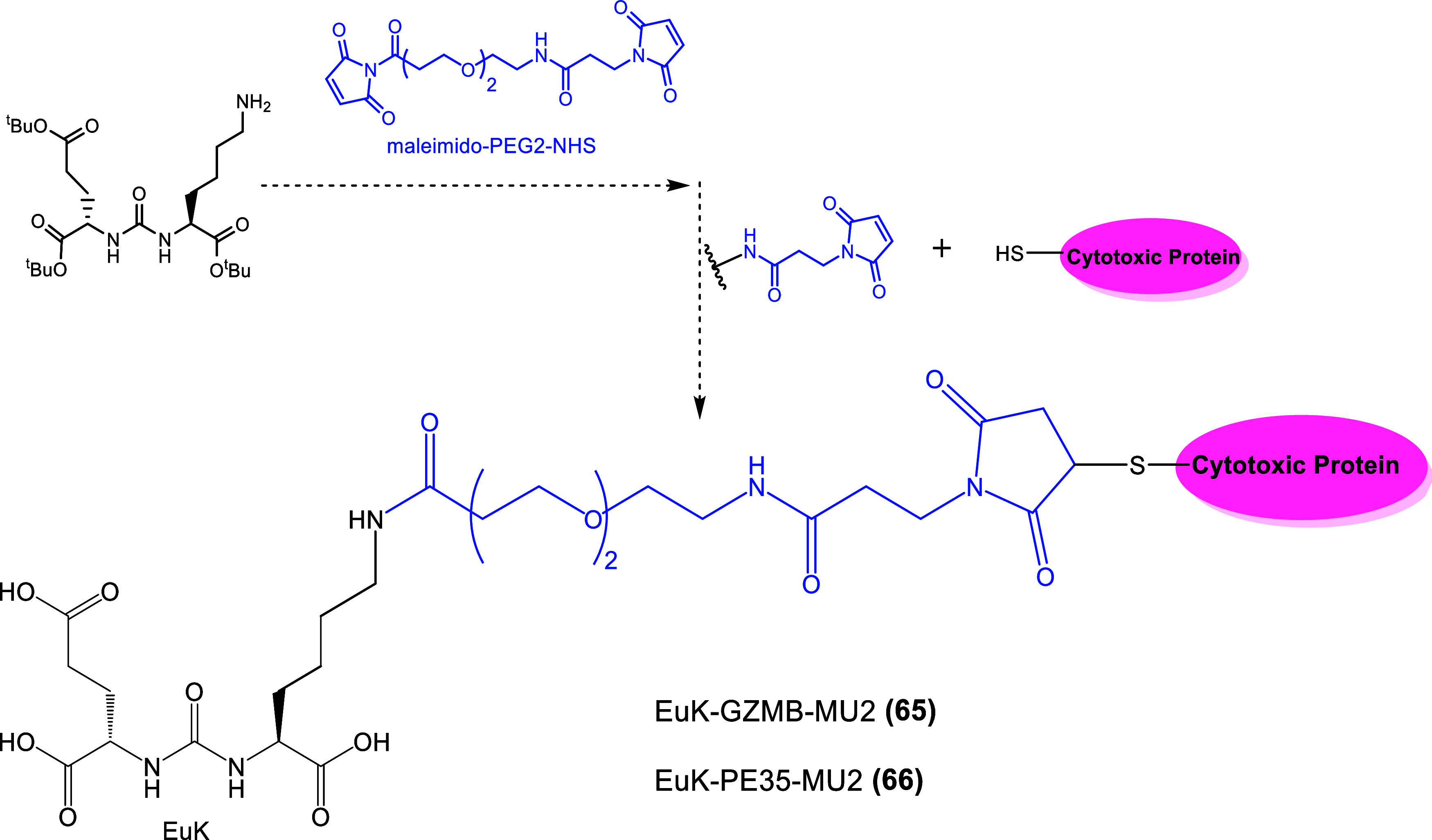
Bioconjugates of EuK with cytotoxic proteins (magenta highlight) GZMB-MU2 (**65**) and PE35-MU2 (**66**). The synthesis was based on the formation of S-protein bonds with the free SH of each protein (EuK: black; linker: blue color).

The IC_50_ values for the two bioconjugates, determined by their enzymatic activity, were **65** (EuK-GZMB-MU2), IC_50_ = 58.7 nM and **66** (EuK-PE35-MU2), IC_50_ = 416 nM. However, despite the lower IC_50_ value, **65** was found to be inactive when incubated with cells. In contrast, **66** was selectively toxic against PSMA-expressing cells showing much lower IC_50_ values in PSMA­(+) cells (PC-3 PIP cells, IC_50_ = 0.9 nM; LNCaP cells, IC_50_ = 0.2 nM; CWR22 Rv1 cells, IC_50_ = 2.2 nM; LAPC4 cells, IC_50_ = 4.5 nM) compared with PSMA(−) cells (Flu-PC-3, IC_50_ = 29.9 nM; DU145, IC_50_ = 24.9 nM).^43^ *In vivo* studies in mice bearing PCa xenografts showed that EuK-PE35-MU2 **65** significantly inhibited the growth of PSMA­(+) PC-3 PIP cells compared with the unmodified PE35-MU2 control but had no effect on the growth of PSMA(−) Flu-PC-3 cells. **65** was injected intratumorally (LNCaP tumor, *D* = 2× 0.8 mg/kg), where it showed >50% average reduction in tumor size at 2 weeks p.i. and >90% reduction in serum PSA levels, while unmodified PE35-MU2 or vehicle controls had no effect. The IV administration of **65** (single dose, *D* = 2 mg/kg) was relatively well tolerated (<15% body weight loss and no animal death).

Higher doses (*D* = 3 mg/kg, *D* = 6 mg/kg) produced ∼15 and 30% body weight loss, respectively, at 1 week post dosing. The optimal dosing (*D* = 2 mg/kg for 4 consecutive days) produced an ∼50% average reduction in LNCaP tumor volume with all treated mice experiencing some degree of antitumor effect at 2 weeks post-therapy, while in the 4-day dosing regimen, no treatment-related deaths occurred, and the treated animals showed a maximum average decline of 15% body weight compared with the 11% when controls were used.^43^

#### Discussion and Concluding Remarks

The application of LTDs carrying cytotoxic drugs is gaining favor in the anticancer drug development because it minimizes side effects of the original cytotoxic drug.^6,8,9,88,89^ PSMA has been the target of choice for the development of LTDs against PCa, due to its vast expression in the PCa cell surface and ability to internalize the carried load inside the cell. In the PSMA-targeting LTDs, the various cytotoxic drugs utilized could block the biochemical processes of the cell or its proliferation, such as SMDCs carrying therapeutic radionuclides, which cause damage to the DNA structure.

Currently, there is limited clinical development regarding PSMA-targeting LTDs in comparison with PSMA-targeting radioligands. There are several factors for that, which are summarized in Table 1. For example, PSMA-targeting radioligands via the exchange of the radionuclide can easily facilitate both diagnosis and therapy (theranostic drugs). Molecular imaging is a powerful tool in drug development; therefore, several groups utilized dyes or radionuclides during LTD preclinical development (mixed design), i.e., **54** (Figure 21) and **57** (Figure 22).

**Table 1:** Summary of Factors That Enable a Faster Clinical Development for PSMA-Targeting Radioligands in Comparison to PSMA-Targeting LTDs

Factor	Radioligands	LTDs
Mechanism of Action	Well-understood for radiation	Complex (requires internalization, release of toxophore)
Development Complexity	Moderate	High (linker, payload, delivery)
Imaging + Patient Selection	Built-in (i.e., PET tracers)	Often lacking
Regulatory Path	Clearer, faster	Slower, more complex
Early Success	Yes (Pluvicto)	Not yet
Pharma Interest	High	Growing, but catching up

The critical questions that need to be answered for this drug category to have vast applications in therapeutic protocols are the following:

- Best payloads
- Best linker designs
- Optimal PSMA binding strategy

Regarding the optimal choice of payload, several factors could apply, among them its availability and production cost. So far, MMAE seems to be the most preferred cytotoxic drug. The characteristics of this cytotoxic moiety are also the optimal criteria of choice for this part of LTDs: (a) it has a very high toxicity, leading to critical side effects, when not targeted; (b) even small amounts are enough to cause the desired effects; (c) it can be easily attached with basic peptide chemistry to the rest of the bioconjugate parts while retaining free its critical parts for biological activity. The second characteristic is very important when cytotoxicity is caused by an intermediate process such as ligand internalization.

What is the optimal linker and binding strategy to attach the toxic payload to the PSMA pharmacophore? The linker length and flexibility or rigidity should minimize the interaction of the toxophore with the binding of the PSMA binding motif to the receptor. A uniform LTD design recipe does not exist, i.e., a longer and more flexible linker is not always the optimal choice to achieve the best affinity, e.g., LTDs **39**, **41**, **47**, and **49** (Figure 18). However, one could optimize a working lead structure aided by computational studies and the success stories of PSMA radioligands.

We should also consider that LTD should release its cytotoxic payload through a chemical or enzymatic cleavage reaction^10^ inside or in proximity to PCa, while at the same time, the linker bond should be stable enough to enable the LTD’s circulation in the body until it reaches its destination, the PCa. Among the various linkers utilized, most researchers design LTDs with an enzymatic cleavage mechanism. The S-S bond, often in the self-immolative form, S-S-(CH_2_)_2_OC­(O), which can be reduced by GSR in the cell endosomes, is such an example. Other commonly used linkers are those activated by the peptidases, e.g., VCitP, the dipeptide Val-Cit in combination with the self-immolative spacer PAB linker, which can be cleaved by the protease cathepsin B. Cathepsin B normally functions within lysosomes at acidic pH 4.6; however, in several cases (e.g., neurological disorders such as Alzheimer’s disease and cancer), it leaks out of the lysosome into the cytosol (pH 7.2), where it retains its enzymatic activity, even in an environment of neutral pH, and activates cell death or inflammation.^155^ For most efficient cleavage, recent studies have suggested alternatives for the peptide VCitP linker, e.g., the EVCitP, which are more stable in mouse plasma.^110^ Other peptide linkers can be activated by cathepsin B in a wide range of pH without being susceptible to cleavage by other cysteine cathepsins, e.g., Z-Nle-Lys-Arg-Amc (Amc = 7-amino-4-methylcoumarin with fluorescent properties), which displayed the advantageous properties of measuring high cathepsin B-specific activity over acidic to neutral pHs and selective cleavage by cathepsin B over other cysteine cathepsins.^155^ Z-Nle-Lys-Arg-AMC has not yet been utilized for the synthesis of bioconjugates but can provide an alternative linker for future studies.

The incorporation of the AG10 analogs (selective oral TTR stabilizer) as a second ligand and the initial good results of the studied trimodal LTDs (PSMA, TTR, and cathepsin B) provide the avenue for additional possibilities. For example, the bimodal SMDCs targeting PSMA/GRPR,^156^ recently developed by several groups for theranostic applications, can lead to the development of LTDs targeting PSMA/GRPR using a toxophore like MMAE. The toxophore can be conjugated either to the PSMA or to the GRPR pharmacophore through a suitable linker, for example, S-S-(CH_2_)_2_OC­(O) and VCitP or EVCitP linker.

The recent success of G202/Mipsagargin (**19**) is a recent example of LTD, which has proceeded into clinical studies. Considering the recent examples of SMDCs presented in this review, we can predict a dramatic increase of LTDs’ approvals in the next years as therapeutic agents targeting PSMA/GCPII.

## Abbreviations Used

ADCs: antibody–drug conjugates
AEs: adverse effects
CCR2: CC chemokine receptor 2
CCL2: CC chemokine ligand 2
CCR5: CC chemokine receptor 5
COM: center of mass
D: dose
HCC: hepatocellular carcinoma
GBM: recurrent/progressive glioblastoma
GCPII: glutamate carboxypeptidase II
GERD: gastroesophageal reflux disease
GR: glutathione reductase
LTDs: ligand-targeted drugs
mCRPC: metastatic castration-resistant prostate cancer
MNLD: maximum nonlethal dose
MTD: maximum tolerated dose
LD: median mortality dose
PCa: prostate cancer
SMDCs: small-molecule drug conjugates
TGI: tumor growth inhibition
TLC: thin layer chromatography
